# The changing views on the evolutionary relationships of extant Salamandridae (Amphibia: Urodela)

**DOI:** 10.1371/journal.pone.0198237

**Published:** 2018-08-01

**Authors:** Michael Veith, Sergé Bogaerts, Frank Pasmans, Sarah Kieren

**Affiliations:** 1 Department of Biogeography, Trier University, Trier, Germany; 2 Waalre, the Netherlands; 3 Department of Pathology, Bacteriology and Avian Diseases, Faculty of Veterinary Medicine, Ghent University, Merelbeke, Belgium; Laboratoire de Biologie du Développement de Villefranche-sur-Mer, FRANCE

## Abstract

The phylogenetic relationships among members of the family Salamandridae have been repeatedly investigated over the last 90 years, with changing character and taxon sampling. We review the changing composition and the phylogenetic position of salamandrid genera and species groups and add a new phylogeny based exclusively on sequences of nuclear genes. *Salamandrina* often changed its position depending on the characters used. It was included several times in a clade together with the primitive newts (*Echinotriton*, *Pleurodeles*, *Tylototriton*) due to their seemingly ancestral morphology. The latter were often inferred as a monophyletic clade. Respective monophyly was almost consistently established in all molecular studies for true salamanders (*Chioglossa*, *Lyciasalamandra*, *Mertensiella*, *Salamandra*), modern Asian newts (*Cynops*, *Laotriton*, *Pachytriton*, *Paramesotriton*) and modern New World newts (*Notophthalmus*, *Taricha*). Reciprocal non-monophyly has been established through molecular studies for the European mountain newts (*Calotriton*, *Euproctus*) and the modern European newts (*Ichthyosaura*, *Lissotriton*, *Neurergus*, *Ommatotriton*, *Triturus*) since *Calotriton* was identified as the sister lineage of *Triturus*. In pre-molecular studies, their respective monophyly had almost always been assumed, mainly because a complex courtship behaviour shared by their respective members. Our nuclear tree is nearly identical to a mito-genomic tree, with all but one node being highly supported. The major difference concerns the position of *Calotriton*, which is no longer nested within the modern European newts. This has implications for the evolution of courtship behaviour of European newts. Within modern European newts, *Ichthyosaura* and *Lissotriton* changed their position compared to the mito-genomic tree. Previous molecular trees based on seemingly large nuclear data sets, but analysed together with mitochondrial data, did not reveal monophyly of modern European newts since taxon sampling and nuclear gene coverage was too poor to obtain conclusive results. We therefore conclude that mitochondrial and nuclear data should be analysed on their own.

## Introduction

Salamandridae is the second speciose family of tailed amphibians. Currently, 21 genera with 118 species are recognized within three sub-families: Pleurodelinae (100 species in 16 genera; we here, in contrast to [1], consider *Liangshantriton* being part of *Tylototriton*, since [2], who introduced this new genus, did not provide a diagnosis [3], and also the AmphibiaChina online database [4] does not list this genus), Salamandrinae (16 species in four genera) and Salamandrininae (2 species in one genus) ([1]; date of access: 10.10.2017). The family has a Holarctic distribution, with the majority of species living in Europe and Asia. Apart from their morphological and ecological variability, Salamandridae are known for an exceptionally broad spectrum of reproductive strategies and breeding behaviours (e.g., [5, 6]). The evolutionary relationships of extant salamandrids were therefore repeatedly subject of phylogenetic research, starting in 1928 with Bolkay’s important analysis of cranial characters [7] and ending in the 21^st^ century with numerous molecular genetic studies and their in part contradicting results (S1 Tab).

In 1928, Bolkay inferred a first schematic—what he called—‘idea’ of the phylogenetic relationships of species of the urodelan family Salamandridae from cranial characters [7]. At that time, tailed amphibians (order Caudata) were subdivided into three suborders: Cryptobranchoidea, Ambystomoidea and Salamandroidea. Within the latter, the family Salamandridae included not only today’s Salamandridae, but also the genera *Proteus*, *Necturus*, *Siren* and *Amphiuma* [8]. Bolkay studied almost all extant salamandrid genera. Only a few years later, Herre combined skeletal and mating behavioural characters to provide a modified view of Bolkay’s phylogenetic tree [8]. Wahlert in 1953 was the first who inferred a Salamandridae phylogeny which included only those genera which are also today subsumed under the family [9]. He compared cloaca, oviduct and egg morphology of most extant genera accepted as monophyletic at that time. Salthe in 1967 was convinced that many features of the mating behaviour of the salamandrid genera were so complex that they had to be treated as synapomorphic characters, allowing the inference of a phylogeny based on these features alone [10]. Combining again morphology and behaviour, and referring to previously published phylogenetic trees, Thorn published a first synthesis in his monograph of the Salamanders of Europe, Asia and Northern Africa [11]. Wake and Özeti introduced completely new characters, namely the morphology of the feeding apparatus [12]. They were the first to apply a variety of strictly phenetic approaches, including character state transformation matrices. In contrast, Arnold only used courtship behaviour to infer his phylogeny of Salamandridae [13], while Naylor specifically studied the vertebral column and the trunk musculature [14]. Freitag published what he called ‘his version’ of the phylogenetic relationships of the salamandrid genera based on morphology, life history and behaviour [15], whereas Laurent was the first who also used dentition of jaws to discuss the phylogenetic relationships of Salamandridae [16]. Combining osteology, external morphology and mating behaviour, Scholz in 1995 inferred two alternative cladograms that differed only in the basal splits of true salamanders [17].

In the same year, Titus and Larson published a first molecular phylogeny of the Salamandridae based on ca. 1,000 base pairs (bp) of fragments of the mitochondrial 12S and 16S rRNA genes [18]. They compared it with a maximum parsimony tree of 44 parsimony informative morphological characters. Adding sequences of the mitochondrial cytochrome *b* gene, Caccone et al. published a phylogenetic tree of five genera in order to elucidate the potential monophyly of *Calotriton* and *Euproctus* [19]. Consensus cladograms published by Sever [20] and Garcia-Paris et al. [21] each provided a synopsis of the current knowledge, based on morphology, allozymes and mtDNA or on molecular data alone, respectively. Steinfartz et al. extended the molecular data of [18] and used Bayesian inference to infer a Salamandridae phylogeny based on ca. 1,800 bp [22]. A further extension in terms of base pairs and species was published by Weisrock et al. [23] in 2006. In the same year, Frost et al. [24] published their large and comprehensive amphibia tree based on mtDNA and ncDNA and applying the POY algorithm of phylogenetic inference. A mitogenomic tree of all genera, mostly represented by multiple species, resulted in the first almost fully resolved molecular tree of the Salamandridae [25]. Some taxa with data for ND1 and ND2 were added to Zhang’s et al. [25] data set by Arntzen et al. [26], which resulted in an almost identical tree. In 2011, Pyron and Wiens published a further all-amphibian tree using available data of three mitochondrial and nine nuclear genes [27]. Pyron extended this dataset in terms of taxon sampling [28]. Finally, Marjanovic and Witzmann added a maximum parsimony analysis of 98 morphological, osteological and behavioural characters [29].

We here review the changing views on the evolutionary relationships of Salamandridae over time, with emphasis on extant taxa. We mainly focus on the relationships of genera. We finally add a new phylogeny based on nuclear DNA only and discuss possible reason for divergence among phylogenies.

## Material and methods

### Review of published phylogenies

We transferred published phylogenies into simplified cladograms (S1 Fig). Extinct taxa are omitted, so classical morphological trees, which often include paleontological data, can be easier compared to molecular phylogenies that have to rely on existing taxa only.

Where possible we adapted former nomenclature to current taxonomy in order to make phylogenies published during the last 90 years comparable. We applied the group names of [25]: True salamanders comprise the genera *Chioglossa*, *Mertensiella*, *Salamandra* and *Lyciasalamandra* (see also [30]). The newts are divided into primitive newts (*Echinotriton*, *Pleurodeles* and *Tylototriton*), New World newts (*Notophthalmus* and *Taricha*), Corsica-Sardinia newts (*Euproctus*), modern European newts (*Calotriton*, *Ichthyosaura*, *Lissotriton*, *Neurergus*, *Ommatotriton* and *Triturus*) and modern Asian newts (*Cynops*, *Laotriton*, *Pachytriton* and *Paramesotriton*). The genus *Salamandrina* does not fit into any of these categories; it is therefore mentioned by its genus name.

Since we focus on the phylogenetic relationships of currently accepted genera, we did not extend our review to papers that specifically studied intra-generic relationships, such as for the genus *Salamandra* (e.g., [22, 31, 32]) or the genus *Triturus sensu lato* (e.g., [33–37]).

### Molecular analyses

We analyzed members of all existing and accepted genera of the family. In detail, we analyzed two species per genus of the modern European Newts and two species each of *Euproctus* and *Calotriton*. For all other genera we analyzed one species per genus. Lacking sequence information for some species was completed by using published sequences from GenBank and by forming composite taxa (e.g., [38, 39]). For hierarchical outgroup rooting we used *Batrachuperus yenyuanensis*, *Andrias davidianus*, *Alytes cisternasii* and *Bufo bufo* (for details see Table 1).

**Table 1:** Samples analyzed for fragments of RAG1, KIAA, SACS and TTN.

Sample	Sample origin/voucher number	Rag1	KIAA	SACS	TTN
***Calotriton asper***	DNA sample ^a^^,^^c^	AY583348.1 ^b^	MH499776	MH499808	MH499834
***Calotriton arnoldi***	SPM 12052414/E70 ^c^	KC665968.1 ^b^	MH499777	MH499830	MH499858
***Chioglossa lusitanica***	MNCN/ADN 81105 ^c^	AY583347.2 ^b^	MH499780	MH499810	MH499835
***Cynops*** **(composite sequence)**	pet trade ^c^	KC165590.1 ^b^ (*C*. *orientalis*)	MH499778 (*C*. *pyrrhogaster*)	MH499826 (*C*. *pyrrhogaster*)	MH499853 (*C*. *pyrrhogaster*)
***Echinotriton andersoni***	legal private breeding stock ^c^	AB856892.2 ^b^	MH499779	MH499827	MH499854
***Euproctus montanus***	MNHN1978.584	MH499799	MH499781	MH499809	MH499836
***Euproctus platycephalus***	MZUF 22303		MH499782	MH499828	MH499855
***Ichthyosaura alpestris***	DNA sample ^a^	MH499800	MH499783	MH499811	MH499837
***Laotriton laoensis***	ZFMK 97218		MH499796		MH499852
***Lissotriton italicus***	DNA sample ^a^	MH499802	MH499785	MH499813	MH499839
***Lissotriton boscai***	MNCN/ADN 68627	MH499801	MH499784	MH499812	MH499838
***Lyciasalamandra fazilae***	DNA sample ^a^^,^^c^	KF645965.1 ^b^	KF645495 ^b^	MH499814	KF645466 ^b^
***Mertensiella caucasica***	ZMADYU 2011/276	MH499803	MH499786	MH499815	MH499840
***Neurergus crocatus***	DNA sample ^a^	MH499804	KF564163	MH499817	MH499842
***Neurergus kaiseri***	DNA sample ^a^	MH499805	KF564174	MH499816	MH499841
***Notophthalmus viridescens***	HLMD-RA-3209 ^c^	AY650134.1 ^b^	MH499787	MH499831	MH499856
***Ommatotrion ophryticus***	ZMADYU 2011/279	MH499806	KF564197.1 ^b^	MH499818	MH499843
***Ommatotriton vittatus***	ZMADYU 2007/53		MH499791		MH499844
***Pachytriton granulosus***	HLMD-RA-3210		MH499797	MH499833	MH499851
***Paramesotriton*** **(composite sequence)**	pet trade	GQ303710.1 ^b^ (*P*. *deloustali*)	MH499788 (*P*. *hongkongensis*)	MH499829 (*P*. *hongkongensis*)	MH499857 (*P*. *hongkongensis*)
***Pleurodeles waltl***	DNA sample ^a^^,^^c^	AJ010258 ^b^	MH499792	MH499819	MH499845
***Salamandra salamandra***	DNA sample ^a^^,^^c^	KC165600 ^b^	KF645481.1 ^b^	MH499820	KF645453.1
***Salamandrina terdigitata***	DNA sample ^a^^,^^c^	JN695274.1 ^b^	MH499793	MH499821	MH499846
***Taricha*** **(composite sequence)**	pet trade ^c^	AY650133.1 ^b^ (*T*. *rivularis*)	MH499789 (*T*. *granulosa*)	MH499832 (*T*. *granulosa*)	MH499859 (*T*. *granulosa*)
***Triturus marmoratus***	DNA sample ^a^^,^^c^	AY583354.1 ^b^	MH499794	MH499822	MH499848
***Triturus anatolicus***	DNA sample ^a^	MH499807	MH499795	MH499823	MH499847
***Tylototriton*** **(composite sequence)**	DNA sample ^a^^,^^c^	KC165601.1 ^b^ (*T*. *asperrimus*)	MH499790 (*T*. *verrucosus*)		MH499860 (*T*. *verrucosus*)
**Outgroup taxa**					
***Alytes cisternasii***	AYA01	MH499798	MH499774	MH499824	MH499849
***Andrias davidianus***		KC165587 ^b^	KC165375 ^b^		KC165646 ^b^
***Batrachuperus yenyuanensis***		HQ902535 ^b^	JN980021 ^b^	JN980048 ^b^	JN980075 ^b^
***Bufo bufo***	DNA sample ^a^^,^^c^	AY583336 ^b^	MH499775	MH499825	MH499850

Taxa used for composite sequences are given in brackets after the respective accession number; sample origin/voucher numbers are given for samples used by us to obtain DNA sequences not available from GenBank.

^a^ DNA sample from own stock or provided by colleagues

^b^ from GenBank

^c^ sample origin for those not obtained from GenBank

For DNA isolation we used the Qiagen Blood and Tissue Kit. Tissue samples were placed in a 1.5 ml microcentrifuge tube. 20 μl proteinase K were added, the vortexed mix was incubated at 56°C until the tissue was completely lysed. 200 μl Buffer AL and 200 μl ethanol (96–100%) were added and the sample was mixed thoroughly by vortexing. The mixture was pipetted into a DNeasy Mini spin column, placed in a 2 ml collection tube and centrifuged at 6,000 x g (8,000 rpm) for 1 min. The DNeasy Mini spin column was placed in a new 2 ml collection tube, 500 μl AW1 buffer were added and centrifuged for 1 min at 6,000 x g (8,000 rpm). Again, the DNeasy Mini spin column was placed in a new 2 ml collection tube, 500 μl AW2 buffer were added and centrifuged for 3 min at 20,000 x g (14,000 rpm) to dry the DNeasy membrane. Finally, the DNeasy Mini spin column was placed in a clean 2 ml microcentrifuge tube and 200 μl AE Buffer were pipetted directly onto the DNeasy membrane. After final incubation at room temperature for 1 min it was centrifuged for 1 min at 6,000 x g (8,000 rpm) for elution.

We sequenced the following genes: Rag1 (primers RAG1F1 and RAG1R1 of [40], KIAA1239 (primers KIAA1239F1 and KIAA1239R1 for PCR and KIAA1239NF1 and KIAA1239NR1 for nested PCR of [40]), SACS (primers SACSF1 and SACSR1 for PCR and SACSNF1 and SACSNR1 for nested PCR of [40]) and TTN (primers TTNF1 and TTNR1 for PCR and TTNNF1 and TTNNR1 for nested PCR of [40]). The process of PCR reactions was the same for each gene fragment: initial denaturation step for 4 min at 94°C, 45 cycles of denaturation for 45 sec at 94°C, primer annealing for 45 sec at 44.9° and elongation for 120 sec at 65°C; final elongation at for 10 min at 65°C. PCR reactions were prepared using the 5Prime Master Mix and PCR products were purified using the High Pure PCR Product Purification Kit of Roche. The Big Dye Terminater (ABI) was used for Sanger reactions for all genes with the following settings: initial melting for 60 sec at 96°C, 25 cycles of denaturation for 10 sec at 96°C, primer annealing for 5 sec at 50°C and extension for 240 sec at 60°C. Single stranded fragments from both directions each were sequenced on an ABI 35000 Genetic Analyzer Serie 2 sequencer using standard protocols.

We automatically aligned the sequences with MAFFT (version 7, [41]) using the iterative refinement method ([42, 43]) and the Needleman-Wunsch algorithm [44]) with default parameter settings. Sequence alignment was done for each gene separately. We build a supermatrix of all single nuclear gene files by concatenating the sequences (to account for potentially conflicting signals of single genes, we also analyzed them separately; methodological details are given in S1 File). Before concatenation we examined our single nuclear datasets for stop codons and excluded them were necessary. We used the package ape [45] and the function cbind in R (version 3.3.1 [46]) for sequence concatenation.

We manually defined 12 subsets: one subset for every single codon position in every nuclear gene (see Table 2). Partition Finder (version 2.1.1, [47]) determined models of nucleotide evolution and the best-fit partitioning schemes of defined subsets, implementing the greedy algorithm and the Akaike information criterion (AIC) for model selection. In a further step, we compared runs with linked and unlinked partitions, in order to find the best way for treating branch lengths. According to the AIC, the model with linked branch lengths across partitions was the best one (AIC value linked: 37341.14, unlinked: 37522.04). The results were used as *a priori* configurations in the phylogenetic tools.

**Table 2:** Best-fitting partitioning schemes of defined subsets with models of nucleotide evolution.

subset	amount of base pairs	best-fitting substitution model
**Rag1 1**^**st**^ **cp**	408	GTR+G
**Rag1 2**^**nd**^ **cp**	408	TVM+I+G
**Rag1 3**^**rd**^ **cp**	408	TVMef+G
**KIAA1239 1**^**st**^ **cp**	273	TVMef+G
**KIAA1239 2**^**nd**^ **cp, TTN 2**^**nd**^ **cp**	540	GTR+I+G
**KIAA1239 3**^**rd**^ **cp, SACS 3**^**rd**^ **cp**	541	GTR+G
**SACS 1**^**st**^ **cp**	270	TIM+G
**SACS 2**^**nd**^ **cp**	270	GTR+I
**TTN 1**^**st**^ **cp**	267	TrN+I
**TTN 3**^**rd**^ **cp**	266	GTR+I

cp = codon position

We performed phylogenetic analyses using Maximum Likelihood (ML) and Bayesian Inference (BI). We applied the selected partitions and substitution models. The ML tree was calculated with RaxML [48], running 2,000 bootstrap replicates using rapid bootstrapping and the greedy algorithm. BI was performed with MrBayes (version 3.2.6; [49, 50]). Two runs were started separately in order to avoid local maxima, each analysis running four independent Markov Chain Monte Carlo (MCMC) analyses with one cold and three heated chains for 10 million generations. We sampled a tree every 1,000^th^ generation with a burn-in of 20%.

## Results

### Review of published phylogenies

All 25 simplified cladograms derived from published phylogenies as well as from our own tree are shown in the supplement (S1 Fig). Our comparison of these cladograms is mainly based on the shifting position of Zhang et al.’s [25] species groups and their respective monophyly/non monophyly (Table 3).

**Table 3:** Presence of tree topologies (referring to [25]) in published Salamandridae phylogenies.

reference	basal position of *Salamandrina*	monophyly of true salamanders	monophyly of primitive newts	monophyly of New World newts	monophyly of modern Asian newts	paraphyly of modern European newts	paraphyly of mountain newts
[7]	no	yes	no	yes	no	no	no
[8]	no	yes	yes	no	no	yes	no
[9]	no	yes	inconclusive	no	no	inconclusive	no
[10]	i.t.s.	inconclusive	inconclusive	no	inconclusive	inconclusive	no
[11]	no	yes	yes	no	no	yes	no
[12]	no	yes	no	yes	yes	no	no
[13]	i.t.s.	inconclusive	inconclusive	no	inconclusive	yes	i.t.s.
[14]	no	i.t.s.	i.t.s.	yes	i.t.s.	i.t.s.	i.t.s.
[15]	no	yes	yes	no	no	yes	no
[16]	no	yes	yes	yes	no	no	no
[17]	no	yes	no	no	yes	no	no
[18] ^a^)	inconclusive	inconclusive	yes	inconclusive	yes	yes	i.t.s.
[19] ^a^)	i.t.s.	i.t.s.	i.t.s.	i.t.s.	i.t.s.	yes	yes
[20]	inconclusive	yes	yes	yes	yes	inconclusive	inconclusive
[21]	inconclusive	yes	yes	yes	yes	inconclusive	no
[22] ^a^)	inconclusive	yes	yes	yes	i.t.s.	yes	yes
[23]	inconclusive	yes	yes	yes	yes	inconclusive	inconclusive
[24]	i.t.s.	i.t.s.	yes	yes	i.t.s.	i.t.s.	i.t.s.
[25] ^a^)	yes	yes	yes	yes	yes	yes	yes
[27] ^a^)	yes	inconclusive	yes	yes	yes	yes	yes
[28] ^a^)	inconclusive	yes	yes	yes	yes	yes	yes
[26] ^a^)	inconclusive	yes	yes	yes	yes	yes	yes
[29]	no	yes	yes	inconclusive	no	no	no
**this study** ^a^)	yes	yes	yes	yes	i.t.s.	no	yes

i.t.s. = insufficient taxon sampling to test this hypothesis.

^a)^ Based on bootstrap support values ≥ 70% and/or Bayesian Posterior Probabilities ≥ 95%

#### The genus *Salamandrina*

Almost all phylogenetic trees based on morphology and behaviour placed *Salamandrina* together with newts, mostly with the primitive newts *Tylototriton* and *Pleurodeles* ([8, 9, 11, 14–17]). Only [12] and [18] morphologically affiliated the genus to the true salamanders based on their phenetic or parsimony analyses of characteristics of the feeding apparatus or other morphological characters, respectively. That *Salamandrina* might belong to neither true salamanders nor newts was first indicated by the mitochondrial tree presented by Titus and Larson [18]. At that time its position within the Salamandridae seemed to be basal, although without sufficient support. Sever and Garcia-Paris acknowledged this unclear position in their consensus trees [20, 21]. Steinfartz et al. were the first to place *Salamandrina* as the sister lineage to all other Salamandridae [22], while Weisrock et al. placed the genus as sister lineage to newts [23]; in both phylogenies support for the respective position was still weak. Significant support for a sister relation of *Salamandrina* and all other Salamandridae was finally established by the tree of Zhang et al. based on complete mitogenomes [25]. The only non-molecular analysis which placed *Salamandrina* as a sister lineage to all newts was Marjanovic and Witzmann [29].

#### True salamanders (*Salamandra*, *Lyciasalamandra*, *Mertensiella* and *Chioglossa*)

Since Bolkay [7], the genera *Salamandra*, *Lyciasalamandra*, *Mertensiella* and *Chioglossa* emerged as a monophylum in almost all analyses, irrespective of the characters used. One exceptions was published by Salthe who includes *Tylototriton* and *Pleurodeles* in this clade since their males capture the females from below with their forelimbs during courtship [10]. Titus and Larson in their analysis of morphological traits included *Salamandrina* in the true salamanders [18]. All molecular phylogenies depicted even the same topology within this clade, with *Salamandra* being the sister lineage of *Lyciasalamandra* and *Chioglossa* being the sister lineage of *Mertensiella*.

The clade of true salamanders was seen as the sister clade to all other Salamandridae by [8, 9, 14, 16, 17: cladogram II, 23, 24, 29]. Divergent position resulted from the fact that other clades were seen basal to all Salamandridae, such as *Euproctus/Calotriton* [7, 10], primitive newts [12], *Salamandrina* [22, 25, 27, 28] or a combined clade of true salamanders and other genera [11, 15, 17: cladogram I, 18: morphological tree]. A completely different position was depicted by Arnold, with *Salamandra* and *Chioglossa* forming a joint clade with *Pleurodeles* within a clade of modern newts [13].

#### Primitive newts (*Echinotriton*, *Pleurodeles* and *Tylototriton*)

Although most studies identified monophyly of Asian and European primitive newts, there are some interesting exceptions. Wake and Özeti placed *Tylototriton*/*Echinotriton* at the basis of the Salamandridae tree, while the next split separated *Pleurodeles* from the remaining taxa [12]. Scholz in his two cladograms made the primitive newts paraphyletic due to a sister relation of *Tylototriton* with *Salamandrina* [17]. Bolkay, Wahlert and Salthe also included primitive newts in the same clade, however with unclear affiliation to each other and/or to other newts within this clade [7, 9, 10]. Wake and Özeti placed European and Asian primitive newts basal at the Salamandridae tree, however not as a monophylum [12]. Starting with the molecular study of Titus and Larson [18] their monophyly was no longer doubted, neither was their independent position within the tree.

#### New World newts (*Notophthalmus* and *Taricha*)

Since molecular data are used to analyse the phylogenetic relationships among salamandrid genera, the sister relationship of *Taricha* and *Notophthalmus* is widely accepted. In contrast, morphological data only rarely inferred this relationship [7, 12, 14, 16]. In most such cases *Taricha* was considered to show far more primitive characters than *Notophthalmus* (e.g., [9, 10, 11, 13, 15, 17]), and Herre even considered the genus *Taricha* paraphyletic [8]; at that time, *T*. *torosa* was considered *Diemytylus torosus* or even *N*. *torosa* by some scientist.

#### Modern Asian newts (*Cynops*, *Laotriton*, *Pachytriton* and *Paramesotriton*)

As with the New World newts, modern Asian newts always emerged as being monophyletic when molecular data were used for phylogenetic reconstruction. Changing affiliations of their genera to various other Salamandridae taxa emerged from non-molecular studies, with no consistent pattern becoming obvious. Among the latter, monophyly of modern Asian newts was only inferred by Wake and Özeti, Naylor (based on an incomplete taxon sampling) and Scholz [12, 14, 17].

#### Corsica-Sardinia newts (*Euproctus*) and modern European newts (*Calotriton*, *Ichthyosaura*, *Lissotriton*, *Neurergus*, *Ommatotriton* and *Triturus*)

The changing phylogenetic relationships within and between these two groups have to be described together, since much of this is related to the changing positions of the genera *Euproctus* and *Calotriton*. Over decades it was taken for granted that today’s *Calotriton asper* was a species of *Euproctus* (we here call this clade *Euproctus sensu lato)*; therefore, most authors did not even consider studying both taxa. Even the first comprehensive molecular study on Salamandridae published by Titus and Larson only included *Euproctus (Calotriton) asper* as the sole representative of the genus [18]. In the same way, *Ichthyosaura*, *Lissotriton*, *Ommatotriton* and *Triturus* were for a long time considered being representatives of the genus *Triturus* (we here call this clade *Triturus sensu lato*) since they shared a complex mating behaviour. Interestingly, Bolkay already identified different species groups within *Triturus* (at that time *Triton*): *Paleotriton* (today’s *Lissotriton* and *Ommatotriton*), *Mesotriton* (today’s *Neurergus* and *Ichthyosaura*) and *Neotriton* (today’s *Triturus*) [7]. However, later authors did not follow his taxonomy and considered all containing species, except *Neurergus*, *a priori* as a monophyletic genus *Triturus*.

Bolkay placed a *Euproctus*/*Calotriton* clade basal in his Salamandridae tree ([7]; this opinion was later on shared by [10]). While *Neurergus* and *Triturus sensu lato* remained monophyletic in the following studies, the position of *Euproctus/Calotriton* changed. Herre and Titus and Larson placed it as sister lineage to *Pachytriton* [8, 18], Wahlert and Thorn inferred a sister relationship with *Notophthalmus* [9, 11], while Wake and Özeti placed *Euproctus/Calotriton* as sister lineage of a clade formed by modern European and New World newts [12]. In the cladogram of Laurent *Euproctus/Calotriton* is the sister lineage of a clade formed by modern Asian and modern European newts [16]. Scholz described the phylogenetic relationship of *Euproctus/Calotriton* to the modern European and the New World newts as being ambiguous, however, within a clade formed by all modern newts [17].

Caccone et al. were the first to hint at reciprocal paraphyly of the genera *Euproctus sensu lato* and *Triturus sensu lato*, although they explicitly stress that their data were not conclusive in establishing non-monophyly of both genera [19]. This was reflected by the consensus trees presented by Sever and Garcia-Paris et al. [20, 21]. At that time the use of the name *Calotriton* for the Pyrenean newts and of *Lissotriton*, *Ichthyosaura* (for a while it was called *Mesotriton* according to Bolkay’s [7] nomenclature), *Ommatotriton* and *Triturus* for subclades within *Triturus sensu lato* had already established. With regard to these species groups, the tree of Steinfartz et al. was poorly resolved [22]; however, they established a close and significant sister-relationship of *Calotriton* and *Triturus*. Although based on far more data, also the tree of Weisrock et al. remained inconclusive in most phylogenetic splits, and even the *Triturus/Calotriton* clade was not sufficiently supported [23]. Finally, it were Zhang et al. who’s phylogenetic tree based on complete mito-genomes showed a clear sister relationship of *Euproctus* to a clade formed by modern Asian and modern European newts (with both constituting monophyletic entities), and a sister relationship of *Calotriton* and *Triturus* [25]. Interestingly, inclusion of information from nuclear genes by Pyron and Wiens and Pyron [27, 28] broke up the well-supported relationships established by [25]).

### New nuclear tree

The total alignment consisted of 3,651 base pairs. Partition Finder (version 2.1.1, Lanfear et al. 2016) determined 10 subsets as the best number of partitions: one for every single codon position in every single nuclear gene, except for the second codon position of KIAA1239 and TTN and the third codon position of KIAA1239 and SACS, which were defined as one subset (see Table 2 for the single subsets in combination with the best-fitting substitution models).

In the four single-gene trees (shown in S1 File) some taxa shifted their position compared to the concatenated tree: (*i*) *Salamandrina* in the Rag1 and TTN trees, (*ii*) *Calotriton* in the Rag1, KIAA and TTN trees and (*iii*) *Ichthyosaura alpestris* in the SACS tree. However, none of these positional changes were sufficiently supported by BPP and bootstrap values, so they do not contradict the phylogenetic information provided by the concatenated tree, which is almost fully resolved. We therefore further on only discuss the concatenated tree.

**Fig 1:**
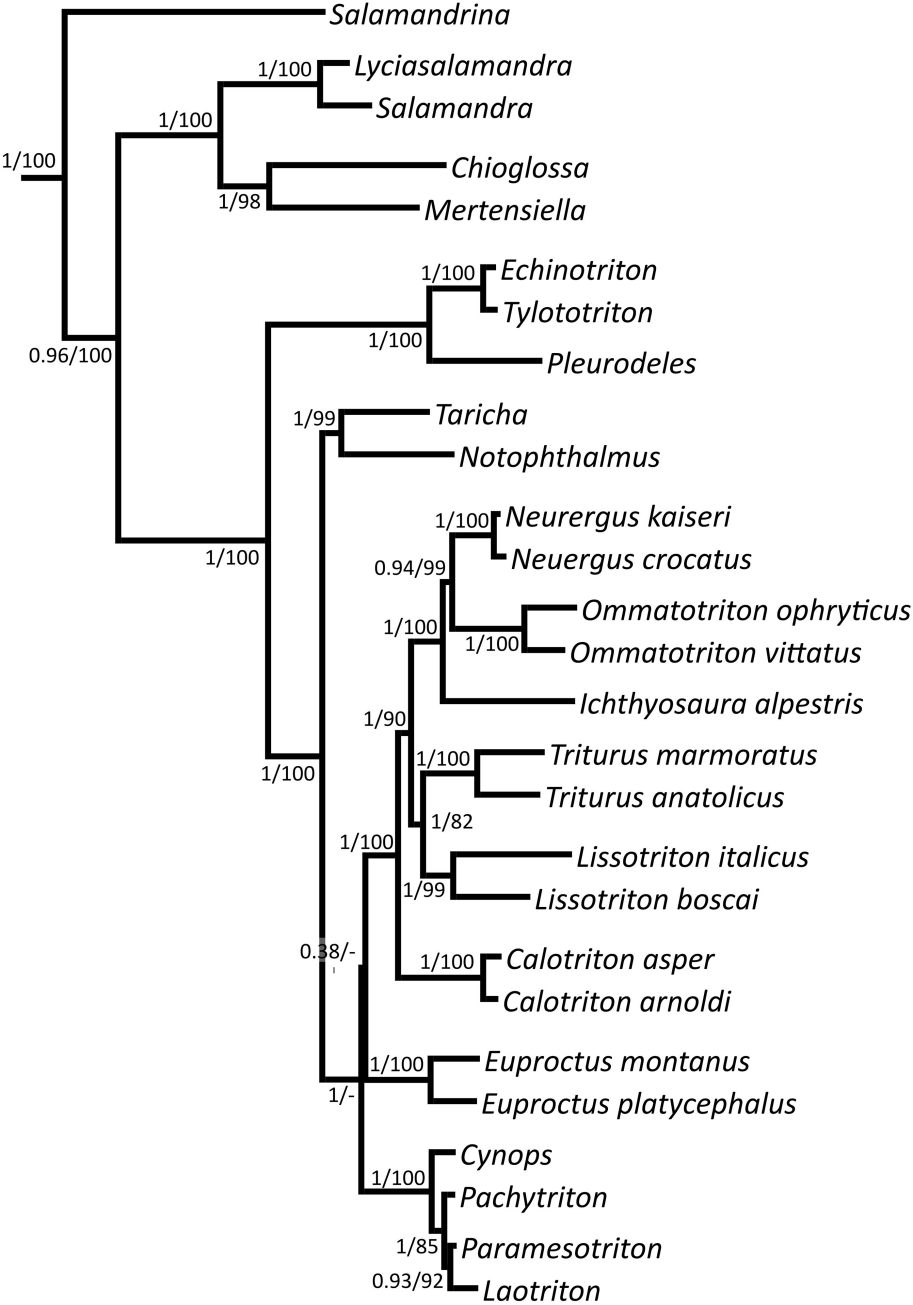
Bayesian tree of Salamandridae based on four nuclear genes. Bayesian posterior probabilities/bootstrap support values for the maximum likelihood tree are given at the nodes.

Bayesian and maximum likelihood trees show identical topologies, with almost all splits within Salamandridae being highly supported (Fig 1; the respective cladogram is given in S1 Fig). The tree topology is almost identical to the tree given by [25]. Differences become obvious within the modern European newts and with the position of *Euproctus*. *Calotriton* is clearly the sister lineage of the modern European newts, so it is no longer sister to the large-bodied newts of the genus *Triturus* as it was inferred by mtDNA. Consequently, *Triturus sensu lato* plus *Neurergus* form a monophylum. Interestingly, within the clade, and in comparison to [25], *Ichthyosaura* and *Lissotriton* changed their positions, with the latter now being the sister lineage of *Triturus*, and *Ichthyosaura* being the sister lineage of the *Ommatotriton/Neurergus* clade (the latter being sufficiently supported only in the ML tree). The entire clade of modern Asian and European newts plus the Corsica-Sardinia newts shows a basal trichotomy, so the position of *Euproctus* within this clade is not clear.

## Discussion

The changing views on the Salamandridae phylogeny were strongly influenced by the data used. Morphological and behavioural characters, used from Bolkay until Marjanovic and Witzmann [7, 29], are usually multigenic and encoded by the nuclear genome. They are prone to homoplastic evolution due to natural and sexual selection. In consequence, their application to phylogenetic reconstruction are potentially misleading since seemingly synapomorphic character states may have evolved through strong selective pressure on single characters or components thereof. A further source of phylogenetic misinterpretation may stem from preconceived opinions concerning the monophyly of taxa. They may have been based on a single, seemingly ‘strong’, character such as a complex mating behaviour, which was considered unlikely to have evolved homoplastically. The dorsal tail projection of males of the genera *Mertensiella* and *Lyciasalamandra* is an outstanding example within the Salamandridae. This hedonic gland of the two genera is situated dorsally on the back of the males right above the base of their tails. It was considered synapomorphic since it only occurs in these two species within the family, and, at the same time, is linked to a complex behaviour of the males performed for female stimulation and courtship synchronization (e.g., [51–53]). As soon as molecular phylogenetic trees hinted at paraphyly of *Mertensiella*, with *Lyciasalamandra* now being the sister genus of *Salamandra*, further characters such as the histological structure of the tail projections of *Mertensiella* and *Lyciasalamandra* [54] were carefully interpreted as supportive of the non-monophyly of the former genus *Mertensiella* (e.g., [30]).

Uncertainties about the plesiomorphic or apomorphic evaluation of single character states were also the reason why some taxa ‘shifted’ through the Salamandridae tree in the course of time. Several authors placed *Salamandrina* in one clade with *Pleurodeles* and *Tylototriton* based on the absence of paired premaxillary bones or a well-developed frontosquamosal arch (e.g., [7, 8]). Later on, these character states were interpreted as plesiomorphic (e.g., [17, 18]) and hence are not informative for phylogenetic reconstruction in a strict cladistics sense. On the other hand, behavioural differences between the two Nearctic genera *Taricha* (e.g., clasping females at their head) and *Notophthalmus* (e.g., clasping females at their entire back) almost always placed them on different branches of most Salamandridae trees based on non-molecular data (for exceptions see [7, 12, 16]). Starting with the first molecular tree of Titus and Larson [18] neither their monophyly nor the single colonization of the Nearctic by the Salamandridae was questioned (e.g., [22, 23, 25].

A further interesting group of changing phylogenetic affiliation are the Sardinia-Corsica newts (*Euproctus*) and the genus *Calotriton*. Both genera are confined to fast running mountain creeks and seem to have adapted their mating behaviour to this challenging habitat independently. Especially the caudal tail capture displayed by males of both genera during courtship, a trait that has been interpreted as an adaptation to fast running waters, was attributed to a common ancestor [10]. All aspects of morphology and behaviour in these two genera were so similar that their monophyly was taken as granted, until Caccone et al. showed a sister relationship of *Calotriton asper* with *Triturus carnifex* based on molecular data ([19]; mitochondrial DNA sequences). Even more, most authors up to Titus and Larson [18] included only one species of *Euproctus sensu lato* (interestingly, [7] was the only to include all three species recognized at his time), making the detection of a potential paraphyly impossible.

Steinfartz et al. were the first to analyse a large number of species of *Triturus sensu lato* together with *Euproctus platycephalus*, *E*. *montanus* and *Calotriton asper* [22]. They found significant support for a sister relation of large bodied newts, genus *Triturus*, with *Calotriton*. All other relationships among modern European newts, including *Euproctus*, were not sufficiently supported. Finally, the mitogenomic approach applied by Zhang et al. resulted in a phylogenetic tree where the positions of *Euproctus* und *Calotriton* were unambiguously resolved: *Calotriton* was the sister genus of *Triturus*, while *Euproctus* was the sister lineage of a large clade formed by modern European and Asian newts [25]. Consequently, the similar mating behaviour of *Euproctus* and *Calotriton*, must have evolved in parallel, which may be indicated by slight differences in the application of caudal capture [55] as outlined by [22]. Alternatively, it evolved from a courtship adapted to lentic habitats back to an ancient caudal capture behaviour which would also have worked in terrestrial mating [10]. Later phylogenetic studies which included sequence data from nine nuclear genes [27, 28] supported a *Calotriton/Triturus* clade, but they remained ambiguous with relation to the position of *Euproctus*. Kieren’s et al. [6] reconstruction of ancient mating behaviour within the Salamandridae is based on the tree topology inferred by [25]. They showed that both *Calotriton* and *Euproctus* evolved from lentic ancestors, hence their behavioural adaptation to lotic habitats evolved convergently and, as shown by their application of the molecular clock, at different times.

Our new tree is the first to be solely based on nuclear genes. It tells a new story of the evolution of the modern European newts, which now again form a monophylum. *Calotriton* is no longer placed within this clade as the sister lineage of *Triturus*. Rather it emerges as the sister lineage of the entire *Triturus sensu lato/Neurergus* clade. *Euproctus* is the sister lineage to the modern European newts; however, branch support is weak, why we consider this relationship as not established. These results may have implications for the evolution of mating behaviour of European and Asian newts. Under the assumption that mating in stagnant water is the ancestral character state (see [6]), the behavioural adaptations of *Euproctus* and *Calotriton* to mating in fast running water may still have evolved convergently. However, we can no longer exclude that this transition occurred only once (as may be implied by Fig 1) in the ancestor of a possible *Euproctus*/*Calotriton*/Modern European newts clade, with the complex mating behaviour of the members of the *Triturus sensu lato/Neurergus* clade being a reversal due to a habitat change back to lentic waters (except for *Neurergus*, who are often found in lotic habitats, where, however, they prefer slow running parts; hence, [56] conclude the lentic behaviour being ancestral). Reconstruction of ancestral character states, the only approach applicable to test these hypotheses, has to combine uncertainties of character state and phylogenetic reconstruction at nodes of interest [57]. Given that the two respective nodes in our nuclear tree are poorly resolved, it is obvious that any reconstruction for them would inevitably become inconclusive. This is why we abstained from applying ancestral character state reconstruction, rather we recommend waiting for a fully resolved nuclear tree before the analyses of [6] are repeated for a nuclear genus level tree. Within the modern European newts, *Triturus* and *Ichthyosaura* changed position compared to the tree of [25], with all nodes being highly supported.

The question remains why [24, 27, 28] did not find a similar change in tree topology, although they studied up to nine (!) nuclear genes compared to only four genes analysed in our study. A re-analysis of the in this respect largest data set ([28]; the other two references used even smaller data sets) offers a simple explanation. Their coverage of nuclear genes for the Salamandridae taxa is extremely poor (on average only 2,003 bp per taxon, ranging from 328 to 5,170 bp), with many salamandrid taxa even not being represented in the nuclear data set included in their >3,300 species tree. Some pairs of taxa do not even share a single homologous base pair; of the modern European newts only *Calotriton asper*, *Triturus cristatus* and *Triturus marmoratus* are included. The nuclear phylogenetic tree for the Salamandridae based in their nuclear genes only therefore remains completely inconclusive (not shown), with many topological peculiarities being mere artefacts of the fragmentary data set. Consequently, and as far as the Salamandridae are concerned, the topology presented by [28] is just an mtDNA tree, with some background noise produced by the few nuclear gene sequences included.

## Conclusion

The changing views on the phylogeny of Salamandridae are due to the changing data used. Taxon sampling, selected characters, preconceived monophyla, as well as misinterpretation of character states as ancestral or derived have generated considerable changes in tree topologies over the last 90 years. The era of molecular phylogenetics seemed to free researches from many of these ambiguities. Nevertheless, topology discordances between molecular phylogenetic trees remained. They may also be due to differential taxon sampling. However, given our new phylogenetic tree based on nuclear data alone, we strongly suggest that nuclear and mitochondrial DNA data should not be merged into one tree. Rather two potentially different stories should be presented. Recent studies on subclades of the Salamandridae also showed that mitochondrial and nuclear data should be treated independently [31, 58]. Even more, the example of the Pyron data [28] shows how misleading a combined analysis of nuclear and mitochondrial data can be, especially when completely unbalanced data sets are combined with respect to taxon and gene coverage.

The mitogenomic tree of [25] and our nuclear tree are largely congruent. However, the few discordant topologies are strongly supported in the respective trees. Such mito-nuclear discordance is now being observed more frequently (e.g., [59]), and their number will continue to grow with an increasing number of nuclear data. Usually, introgression of the mitochondrial genome following hybridization, eventually supported by a selective advantage of the spreading mitochondrial genome, is invoked as an explanation (e.g., [60]). Whether mitochondrial introgression is responsible for the observed discordance between mitochondrial and nuclear Salamandridae trees remains open. Interestingly, the observed discordances only concern European newts, with taxa being involved which, due to their geographic distribution, may in fact have come into contact for hybridization. However, according to molecular clock estimates [6, 25]) such introgression events must have occurred millions of years ago. Further studies have to show whether in fact ancient introgression can be made responsible for the observed mito-nuclear discordance, or if other mechanisms of horizontal gene transfer among genera must be invoked.

## Supporting information

S1 Tab Characters used to reconstruct the phylogeny of Salamandridae. (PDF)

S1 Fig Cladogramms of Salamandridae phylogenies. (PDF)

S1 File Phylogenetic inference of the four nuclear genes. (PDF)

## References

[1] Frost DR. 2017 Amphibian species of the world: an online reference. Version 6.0. [accessed 2017 Oct 10]. Database: American Museum of Natural History, New York, USA. http://research.amnh.org/herpetology/amphibia/index.html.

[2] Fei L, Ye C-Y, Jiang J-P. Colored atlas of Chinese amphibians and their distributions. 2dn ed Sichuan Publishing House of Science & Technology; 2012.

[3] AmphibiaWeb. 2017 [accessed 2017 Oct 10]. Database: University of California, Berkeley, CA, USA. Available from: http://amphibiaweb.org.

[4] AmphibiaChina. The database of Chinese amphibians. 2015 [accessed 2017 Oct 10]. Database. Available from: http://www.amphibiachina.org.

[5] Sparreboom M. Salamanders of the Old World The salamanders of Europe, Asia and northern Africa. 1^st^ edition Zeist: KNNV Publishing; 2014.

[6] Kieren S, Sparreboom M, Hochkirch A, Veith M. A biogeographic and ecological perspective to the evolution of reproductive behaviour in the family Salamandridae. Mol Phyl Evol. 2018; 121: 98–109. 10.1016/j.ympev.2018.01.006 29330138

[7] Bolkay SJ. Die Schädel der Salamandrinen, mit besonderer Rücksicht auf ihre systematische Bedeutung. Z Anat Entw-Gesch (I Abt). 1928; 86: 259–319.

[8] Herre W. Die Schwanzlurche der mitteleocänen (oberlutetischen) Braunkohle des Geiseltales und die Phylogenie der Urodelen unter Einschluß der fossilen Formen. Zoologica (Stuttgart). 1935; 33: 1–85.

[9] von Wahlert G. Eileiter, Laich und Kloake der Salamandriden. Zool Jb Anat. 1953; 73: 276–324.

[10] Salthe SN. Courtship patterns and the phylogeny of the urodeles. Copeia. 1967: 100–117.

[11] Thorn R. Les Salamandres d’Europe d’Asie et d’Afrique du Nord. Paris: Editions Paul Lechevalier; 1968.

[12] Wake DB, Özeti N. Evolutionary relationships in the family Salamandridae. Copeia. 1969: 124–137.

[13] Arnold SJ. The evolution of courtship behavior in salamanders. PhD thesis. University of Michigan. 1972.

[14] Naylor BG. The systematics of fossil and recent salamanders, with special reference to the vertebral column and trunk musculature. PhD thesis, University of Alberta, Edmonton. 1978.

[15] Freitag GE. Über morphologische Eigenheiten und die phyletische Stellung der ostasiatischen Wassermolchgattung *Pachytriton*. Vert Hung. 1982; 21: 127–129.

[16] Laurent RF. Sous-Ordre des Salamandroidea In: Grasse P-P, Delsol M, editors. Traite de Zoologie 14 Paris: Masson; 1986 pp. 636–645.

[17] Scholz KP. Zur Stammesgeschichte der Salamandridae Gray, 1825. Eine kladistische Analyse anhand von Merkmalen aus Morphologie und Balzverhalten. Acta Biol Benrodis. 1995; 7: 25–75.

[18] Titus TA, Larson A. A molecular phylogenetic perspective on the evolutionary radiation of the salamander family Salamandridae. Syst Biol. 1995, 44: 125–151.

[19] Caccone A, Milinkovitch MC, Sbordoni V, Powell JR. Mitochondrial DNA rates and biogeography in European newts (genus *Euproctus*). Syst Biol 1997; 46: 126–144. 11975350 10.1093/sysbio/46.1.126

[20] Sever DM. 2003 Reproductive biology and phylogeny of Urodela. (Reproductive biology and phylogeny, vol 1). Enfield, NH: Science Publishers, ebrary Inc.

[21] Garcia-Paris M, Montori A, Herrero P. Fauna Iberica. Vol. 24. Amphibia. Lissamphibia. Museo Nacional de Ciencias Naturales, Consejo Superior de Investigaciones Cientificas, Madrid; 2004.

[22] Steinfartz S, Vicario S, Arntzen JW, Caccone A. A Bayesian approach on molecules and behavior: reconsidering phylogenetic and evolutionary patterns of the Salamandridae with emphasis on *Triturus* newts. J Exp Zool (Mol Dev Evol). 2006; 308B: 139–162. 10.1002/jez.b.21119 16969762

[23] Weisrock DW., Papenfuss TJ, Macey JR, Litvinchuk SN, Polymeni R, Ugurtas IH, et al A molecular assessment of phylogenetic relationships and lineage accumulation rates within the family Salamandridae (Amphibia, Caudata). Mol Phyl Evol. 2006; 41: 368–383. 10.1016/j.ympev.2006.05.008 16815049

[24] Frost DR, Grant T, Faivovich J, Bain RH, Haas A, Haddad CFB, et al The amphibian tree of life. Bull Am Mus Nat Hist. 2006; 297: 1–370.

[25] Zhang P, Papenfuss TJ, Wake MH, Qu L, Wake DB. Phylogeny and biogeography of the family Salamandridae (Amphibia: Caudata) inferred from complete mitochondrial genomes. Mol Phyl Evol. 2008; 49: 586–597. 10.1016/j.ympev.2008.08.020 18801447

[26] Arntzen JW, Beukema W, Galis F, Ivanović A. Vertebral number is highly evolvable in salamanders and newts (family Salamandridae) and variably associated with climatic parameters. Contrib Zool 2015; 84: 85–113

[27] Pyron RA., Wiens JJ. A large-scale phylogeny of Amphibia including over 2800 species, and a revised classification of caecilians. Mol Phyl Evol. 2011; 61: 543–583. 10.1016/j.ympev.2011.06.012 21723399

[28] Pyron RA. Biogeographic analysis reveals ancient continental vicariance and recent oceanic dispersal in amphibians. Syst Biol 2014; 63: 779–797. 10.1093/sysbio/syu042 24951557

[29] Marjanović D, Witzmann F. An extremely peramorphic newt (Urodela: Salamandridae: Pleurodelini) from the Latest Oligocene of Germany, and a new phylogenetic analysis of extant and extinct salamandrids. PLoS ONE. 2015; 10(9): e0137068 10.1371/journal.pone.0137068 26421432 PMC4589347

[30] Veith M, Steinfartz S, Zardoya R, Seitz A, Meyer A. A molecular phylogeny of “true” salamanders (family Salamandridae) and the evolution of terrestriality of reproductive modes. J Zool Syst Evol Res. 1998; 36: 7–16.

[31] Vences M, Sanchez E, Hauswaldt JS, Eikelmann D, Rodríguez A, Carranza S, et al Nuclear and mitochondrial multilocus phylogeny and survey of alkaloid content in true salamanders of the genus *Salamandra* (Salamandridae). Mol Phyl Evol. 2014; 73: 208–216. 10.1016/j.ympev.2013.12.009 24412216

[32] Rodriguez R, Burgon J, Lyra M, Irisarri I, Baurain D, Blaustein L, et al Inferring the shallow phylogeny of true salamanders (*Salamandra*) by multiple phylogenomic approaches. Mol Phyl Evol. 2017; 115: 16–26. 10.1016/j.ympev.2017.07.009 28716741

[33] Rafinski J, Arntzen JW. Biochemical systematics of the Old World newts, genus *Triturus*: allozyme data. Herpetologica 1987; 43: 446–457.

[34] Giacoma C., Balletto E. Phylogeny of the salamandrid genus *Triturus*. Boll Zool; 1988 55: 337–360.

[35] Arntzen JW, Sparreboom M. A phylogeny for the Old World newts, genus *Triturus*: biochemical and behavioural data. J Zool. 1989; 219: 645–664.

[36] Macgregor HC, Sessions SK, Arntzen JW. An integrative analysis of phylogenetic relationships among newts of the genus *Triturus* (family Salamandridae), using comparative biochemistry, cytogenetics and reproductive interactions. J Evol Biol. 1990; 3: 329–373.

[37] Wielstra B, Arntzen JW. Unraveling the rapid radiation of crested newts (*Triturus cristatus* superspecies. using complete mitogenomic sequences. BMC Evol Biol. 2011; 11: 162. 21672214 10.1186/1471-2148-11-162 PMC3224112

[38] Campbell V, Lapointe F-J. Retrieving a mitogenomic mammal tree using composite taxa. Mol Phyl Evol. 2011; 58: 149–156. 10.1016/j.ympev.2010.11.017 21134474

[39] Diallo AB, Lapointe F-J, Makarenkov V. A new effective method for estimating missing values in the sequence data prior to phylogenetic analysis. Evol Bioinform. 2006; 2: 127–135. PMC2674658 19455216

[40] Shen X-X, Liang D, Zhang P. The development of three long universal nuclear protein-coding locus markers and their application to osteichthyan phylogenetics with nested PCR. PLoS ONE. 2012; 7: 1–11. 10.1371/journal.pone.0039256 PMC3375249 22720083

[41] Katoh K, Standley DM. MAFFT multiple sequence alignment software version 7: improvements in performance and usability. Mol Biol Evol 2013; 30: 772–780. 10.1093/molbev/mst010 23329690 PMC3603318

[42] Berger MP, Munson PJ. A novel randomized iterative strategy for aligning multiple protein sequences. Bioinformatics, 1991; 7: 479–484. 10.1093/bioinformatics/7.4.479 1747779

[43] Gotoh O. Optimal alignment between groups of sequences and its application to multiple sequence alignment. Comput Appl Biosci 1993; 9: 361–370. 8324637 10.1093/bioinformatics/9.3.361

[44] Needleman SB, Wunsch CD. A general method applicable to the search for similarities in the amino acid sequence of two proteins. J Mol Biol. 1970; 48: 443–453. 5420325 10.1016/0022-2836(70)90057-4

[45] Paradis E, Claude J., Strimmer K. APE. Analyses of Phylogenetics and Evolution in R language. Bioinformatics. 2004; 20: 289–290. 14734327 10.1093/bioinformatics/btg412

[46] R Core Team. R: A language and environment for statistical computing. R Foundation for Statistical Computing, Vienna, Austria; 2016.

[47] Lanfear R, Frandsen PB, Wrigt AM, Senfeld T, Calcott B. PartitionFinder2: new methods for selecting partitioned models of evolution for molecular and morphological phylogenetic analyses. Mol Biol Evol. 2016; 34: 772–773. 10.1093/molbev/msw260 28013191

[48] Stamatakis A. RAxML version 8: A tool for phylogenetic analysis and post-analysis of large phylogenies. Bioinformatics. 2014; 30: 1312–1313. 10.1093/bioinformatics/btu033 24451623 PMC3998144

[49] Ronquist F, Huelsenbeck JP. MrBayes 3: Bayesian phylogenetic inference under mixed models. Bioinformatics. 2003; 19: 1572–1574. 12912839 10.1093/bioinformatics/btg180

[50] Ronquist F, Teslenko M, van der Mark P, Ayres DL, Darling A, Höhna S, et al MrBayes 3.2: Efficient Bayesian phylogenetic inference and model choice across a large model space. Syst Biol 2012; 61: 539–542. 10.1093/sysbio/sys029 22357727 PMC3329765

[51] Rehberg F. Nachtrag zu ‘*Mertensiella luschani*, der Lykische Salamander‘ in herpetofauna 9, Seite 16. Herpetofauna 1981; 10: 23.

[52] Klewen R. Die Landsalamander Europas. 2nd ed Ziemsen Verlag, Stuttgart; 1991.

[53] Schultschik G. Zur Fortpflanzungsbiologie von *Mertensiella caucasica*. Abh Ber Naturkd Magdeburg. 1994; 17: 163–175.

[54] Sever DM, Sparreboom M, Schultschik G. The dorsal tail tubercle of *Mertensiella caucasica* and *M*. *luschani* (Amphibia: Salamandridae). J Morphol. 1997; 232: 93–105. 10.1002/(SICI)1097-4687(199704)232:1<93::AID-JMOR6>3.0.CO;2-P 29852686

[55] Thiesmeier B, Hornberg C. Zur Fortpflanzung sowie zum Paarungsverhalten der Gebirgsmolche, Gattung *Euproctus* (Gené), im Terrarium, unter besonderer Berücksichtigung von *Euproctus* asper (Dugès, 1852). Salamandra. 1990; 26: 63–82.

[56] Steinfartz S, Hwand U, Tautz D, Veith M. Molecular phylogeny of the salamandrid genus *Neurergus* within the Salamandridae: Evidence for an intrageneric switch of reproductive biology. Amph Rept. 2002; 23: 419–431.

[57] Pagel M, Meade A, Barker D. Bayesian estimation of ancestral character states on phylogenies. Syst Biol. 2004; 53: 673–684. 10.1080/10635150490522232 15545248

[58] Veith M, Göçmen B, Sotiropoulos K, Kieren S, Godmann O, Steinfartz S. Seven at one blow: The origin of major lineages of the viviparous Lycian salamanders (*Lyciasalamandra*) was triggered by a single paleo-historic event. Amph Rept. 2016, 37: 373–387.

[59] Toews DPL, Brelsford A. The biogeography of mitochondrial and nuclear discordance in animals. Mol Ecol. 2012; 16: 3907–3930. 10.1111/j.1365-294X.2012.05664.x 22738314

[60] Ballard JW, Whitlock MC. The incomplete natural history of mitochondria. Mol Ecol 2004; 13: 729–744. 15012752 10.1046/j.1365-294x.2003.02063.x

