# Supplementary material for: The changing views on the evolutionary relationships of extant Salamandridae (Amphibia: Urodela)
Source: PLoS One. 2018 Aug 1;13(8):e0198237. doi: 10.1371/journal.pone.0198237 (PMC6070172; doi:10.1371/journal.pone.0198237)
Supplement: S1 Tab — (PDF) [file pone.0198237.s001.pdf]

S1 Tab. Characters used to reconstruct the phylogeny of Salamandridae.

reference	osteology	dentition	morphology (misc.)	behaviour	life history	cytology	mtDNA (*partim*)	mito-genomes	ncDNA (*partim*)	consensus trees
**Bolkay (1928)**	x									
**Herre (1935)**	(x)		(x)	( )	( )					
**Wahlert (1953)**	( )		(x)							
**Salthe (1967)**				(x)						
**Thorn (1968)**	x			x						(x)
**Wake and Özeti (1969)**	x		x							
**Arnold (1972)**				x						
**Naylor (1978)**	x		x	( )						
**Freitag (1982)**	x		x	x	x					
**Laurent (1986)**	x	x	x	x		x				
**Scholz (1995)**	x	x	(x)	( )						
**Titus and Larson (1995)**	x		x				x			
**Caccone (1997)**							x			
**Sever (2003)**										( )
**Garcia-Paris et al. (2004)**										( )
**Steinfartz et al. (2006)**							x			
**Frost et al. (2006)**							x		x	
**Weisrock et al. (2006)**							x			
**Zhang et al. (2008)**								x		
**Pyron and Wiens (2011)**							x		x	
**Pyron (2014)**							x		x	
**Arntzen et al. (2015)**							x	x		

**Marjanovic and Witzmann (2015)**	x		x	x						
**this study**									x	

x = own data, ( ) = data from other sources, (x) = own data and data from other sources.

References

Arnold SJ. The evolution of courtship behavior in salamanders. PhD thesis. University of Michigan. 1972.

Arntzen JW, Beukema W, Galis F, Ivanović A. Vertebral number is highly evolvable in salamanders and newts (family Salamandridae) and variably associated with climatic parameters. Contrib Zool 2015; 84: 85-113

Bolkay SJ. Die Schädel der Salamandrinen, mit besonderer Rücksicht auf ihre systematische Bedeutung. Z Anat Entw-Gesch (I Abt). 1928; 86: 259-319.

Caccone A, Milinkovitch MC, Sbordoni V, Powell JR. Mitochondrial DNA rates and biogeography in European newts (genus *Euproctus*). Syst Biol 1997; 46: 126–144.

Freitag GE. Über morphologische Eigenheiten und die phyletische Stellung der ostasiatischen Wassermolchgattung *Pachytriton*. Vert Hung. 1982; 21: 127-129.

Frost DR, Grant T, Faivovich J, Bain RH, Haas A, Haddad CFB, De Sá RO, Channing A, Wilkinson M, Donnellan SC, Raxworthy CJ, Campbell JA, Blotto BL, Moler P, Drewes RC, Nussbaum RA, Lynch JD, Green DM, Wheeler WC. The amphibian tree of life. Bull Am Mus Nat Hist. 2006; 297: 1–370.

Garcia-Paris M, Montori A, Herrero P. Fauna Iberica. Vol. 24. Amphibia. Lissamphibia. Museo Nacional de Ciencias Naturales, Consejo Superior de Investigaciones Cientificas, Madrid; 2004.

Herre W. Die Schwanzlurche der mitteleocänen (oberlutetischen) Braunkohle des Geiseltales und die Phylogenie der Urodelen unter Einschluß der fossilen Formen. Zoologica (Stuttgart). 1935; 33: 1–85.

Laurent RF. Sous-Ordre des Salamandroidea. In: Grasse P-P, Delsol M, editors. Traite de Zoologie 14. Paris: Masson; 1986. pp. 636-645.

Marjanović D, Witzmann F. An extremely peramorphic newt (Urodela: Salamandridae: Pleurodelini) from the Latest Oligocene of Germany, and a new phylogenetic analysis of extant and extinct salamandrids. PLoS ONE. 2015; 10(9): e0137068.

Naylor BG. The systematics of fossil and recent salamanders, with special reference to the vertebral column and trunk musculature. PhD thesis, University of Alberta, Edmonton. 1978.

Pyron RA. Biogeographic analysis reveals ancient continental vicariance and recent oceanic dispersal in amphibians. Syst Biol 2014; 63: 779–797.

Pyron RA., Wiens JJ. A large-scale phylogeny of Amphibia including over 2800 species, and a revised classification of caecilians. Mol Phyl Evol. 2011; 61: 543–583.

Salthe SN. Courtship patterns and the phylogeny of the urodeles. Copeia. 1967: 100–117.

Scholz KP. Zur Stammesgeschichte der Salamandridae Gray, 1825. Eine kladistische Analyse anhand von Merkmalen aus Morphologie und Balzverhalten. Acta Biol Benrodis. 1995; 7: 25–75.

Sever DM. 2003 Reproductive biology and phylogeny of Urodela. (Reproductive biology and phylogeny, vol 1). Enfield, NH: Science Publishers, ebrary Inc.

Steinfartz S, Vicario S, Arntzen JW, Caccone A. A Bayesian approach on molecules and behavior: reconsidering phylogenetic and evolutionary patterns of the Salamandridae with emphasis on *Triturus* newts. J Exp Zool (Mol Dev Evol). 2006; 308B: 139–162.

Thorn R. Les Salamandres d’Europe d’Asie et d’Afrique du Nord. Paris: Editions Paul Lechevalier; 1968.

Titus TA, Larson A. A molecular phylogenetic perspective on the evolutionary radiation of the salamander family Salamandridae. Syst Biol. 1995, 44: 125-151.

Wahlert G von. Eileiter, Laich und Kloake der Salamandriden. Zool Jb Anat. 1953; 73: 276-324.

Wake DB, Özeti N. Evolutionary relationships in the family Salamandridae. Copeia. 1969: 124-137.

Weisrock DW., Papenfuss TJ, Macey JR, Litvinchuk SN, Polymeni R, Ugurtas IH, Zhao, E, Jowkar H, Larson A. A molecular assessment of phylogenetic relationships and lineage accumulation rates within the family Salamandridae (Amphibia, Caudata). Mol Phyl Evol. 2006; 41: 368–383.

Zhang P, Papenfuss TJ, Wake MH, Qu L, Wake DB. Phylogeny and biogeography of the family Salamandridae (Amphibia: Caudata) inferred from complete mitochondrial genomes. Mol Phyl Evol. 2008; 49: 586–597.
