# Supplementary material for: The changing views on the evolutionary relationships of extant Salamandridae (Amphibia: Urodela)
Source: PLoS One. 2018 Aug 1;13(8):e0198237. doi: 10.1371/journal.pone.0198237 (PMC6070172; doi:10.1371/journal.pone.0198237)
Supplement: S1 Fig — (PDF) [file pone.0198237.s002.pdf]

Western Palearctic Salamandridae
Eastern Palearctic Salamandridae
Nearctic Salamandridae
Other families

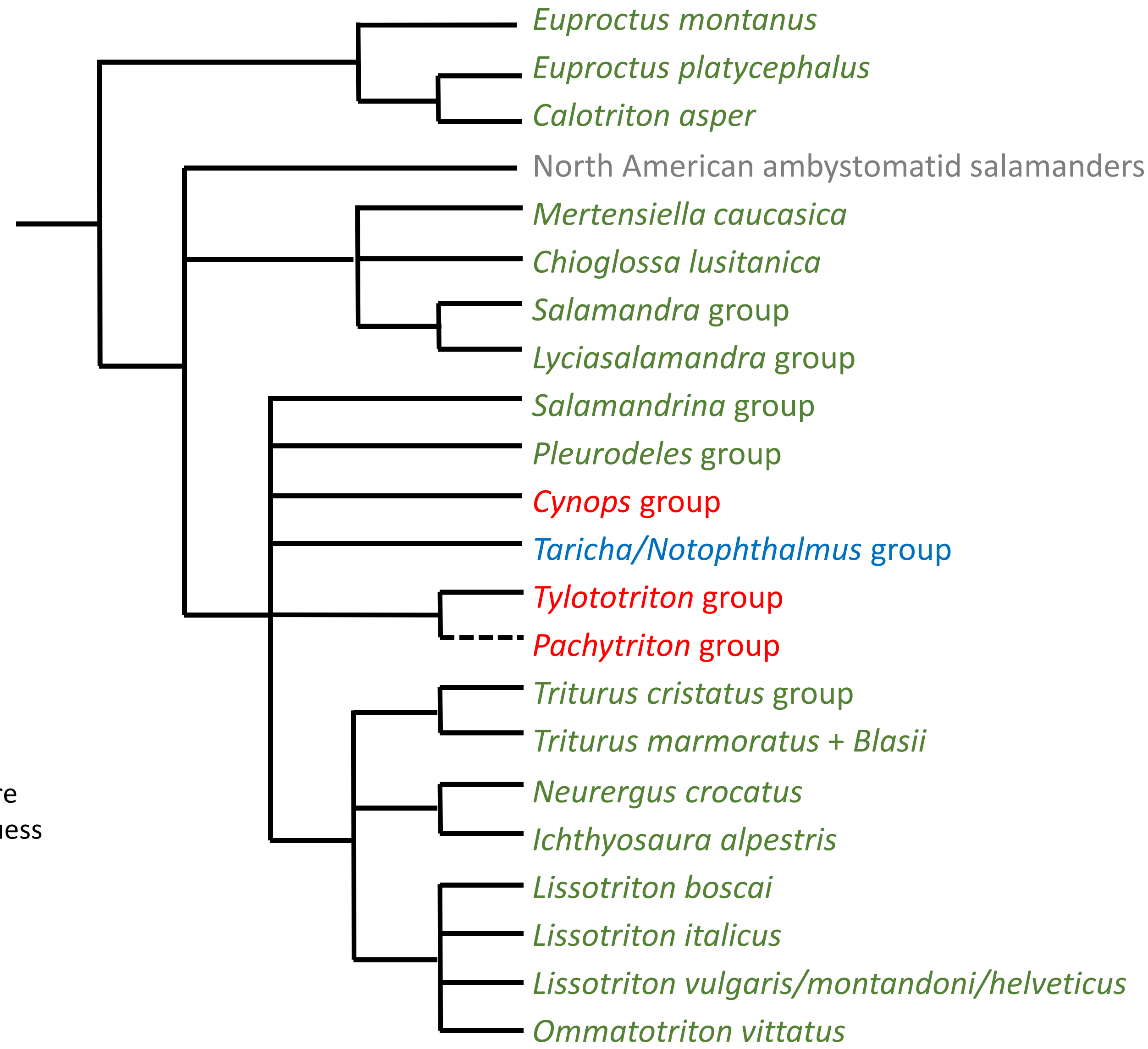

Bolkay (1928):

- Cranial characters
- He did not study *Pachytriton*, therefore his placement of the genus is just a guess

Western Palearctic Salamandridae
Eastern Palearctic Salamandridae
Nearctic Salamandridae
Other families

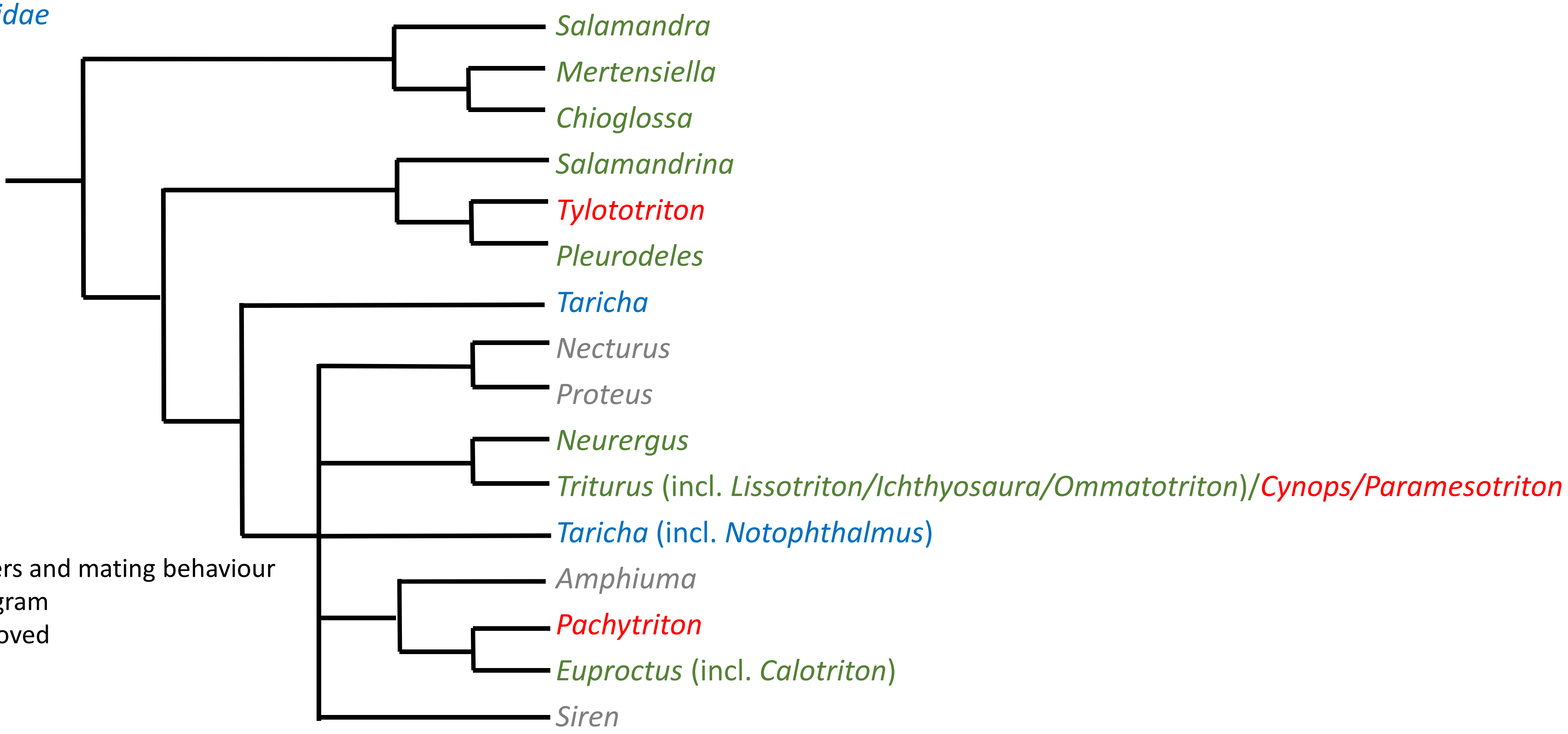

Herre (1935):

- Skeletal characters and mating behaviour
- Simplified cladogram
- Extinct taxa removed

Western Palearctic Salamandridae
Eastern Palearctic Salamandridae
Nearctic Salamandridae

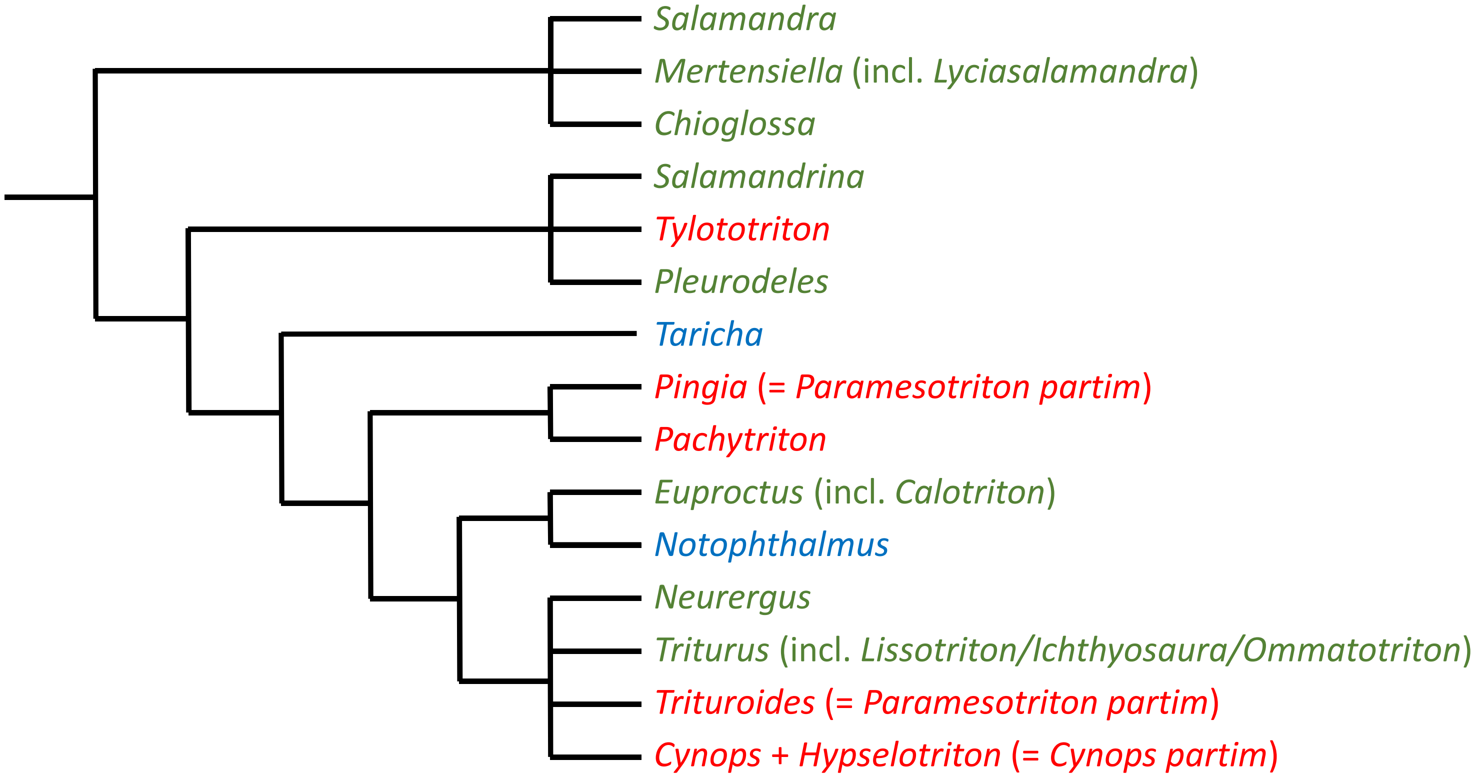

Wahlert (1953):

- Morphology (cloaca, oviduct, eggs)
- *Trituroides* = *Paramesotriton* partim
- *Pingia* = *Pachytriton* partim
- *Hypselotriton* = *Cynops* partim
- He did not study *Pachytriton*, *Pingia* and *Paramesotriton*, so his placement of these genera is just a guess.

Western Palearctic Salamandridae
Eastern Palearctic Salamandridae
Nearctic Salamandridae

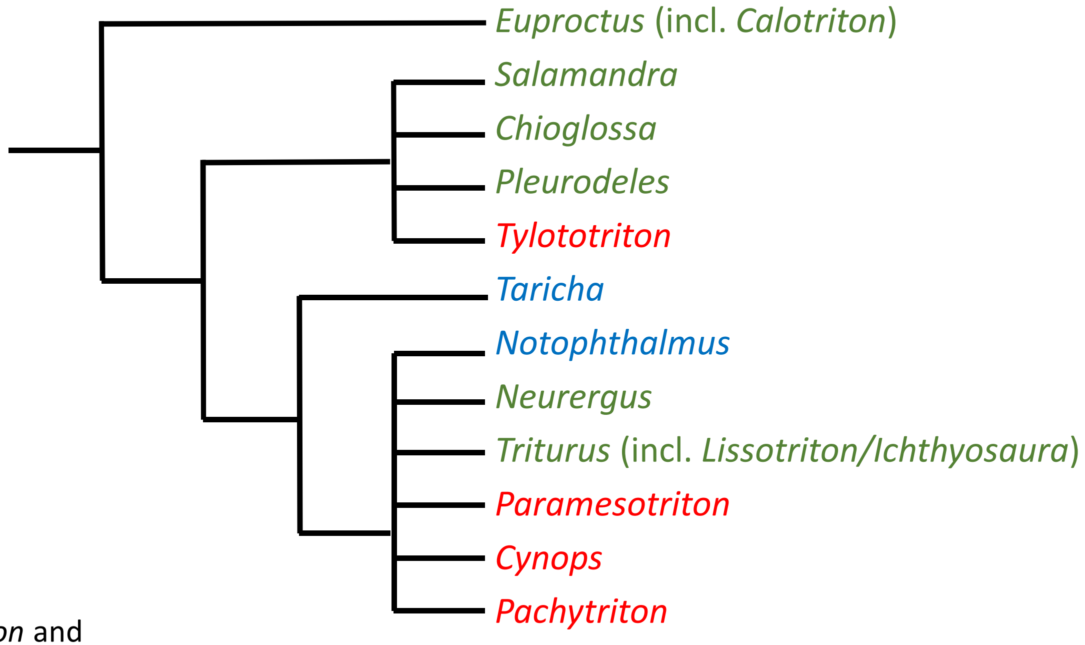

Salthe (1967):

- Reconstruction of Titus and Larson (1995)
- Courtship behaviour
- Courtship behaviour of *Neurergus*, *Pachytriton* and *Paramesotriton* was not described; their close relationships to other taxa are just assumed by him.

Western Palearctic Salamandridae
Eastern Palearctic Salamandridae
Nearctic Salamandridae
Other families

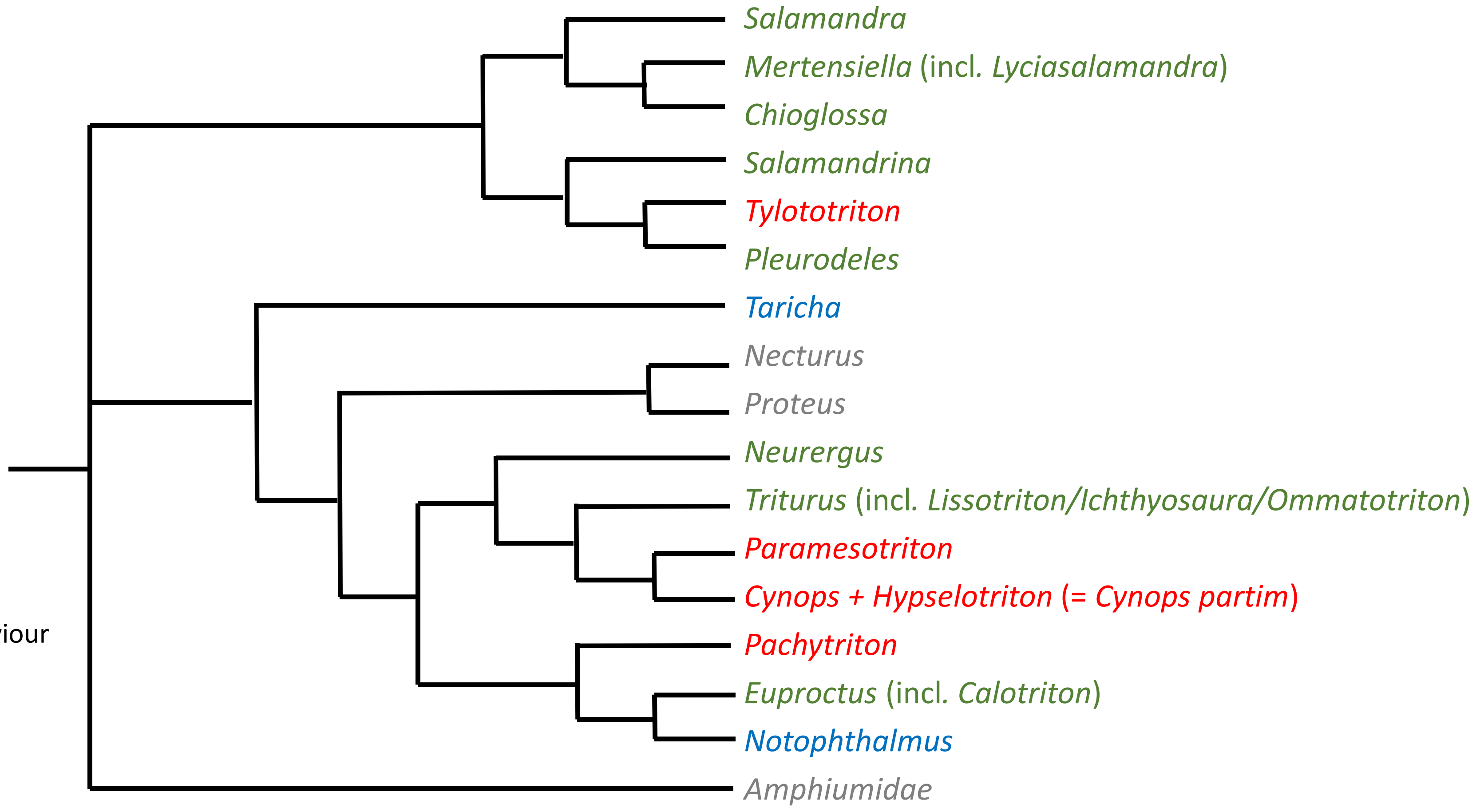

Thorn (1968):

- Salamandroidea
- Morphology and mating behaviour
- Simplified cladogram
- Extinct taxa removed

Western Palearctic Salamandridae
Eastern Palearctic Salamandridae
Nearctic Salamandridae
Other families

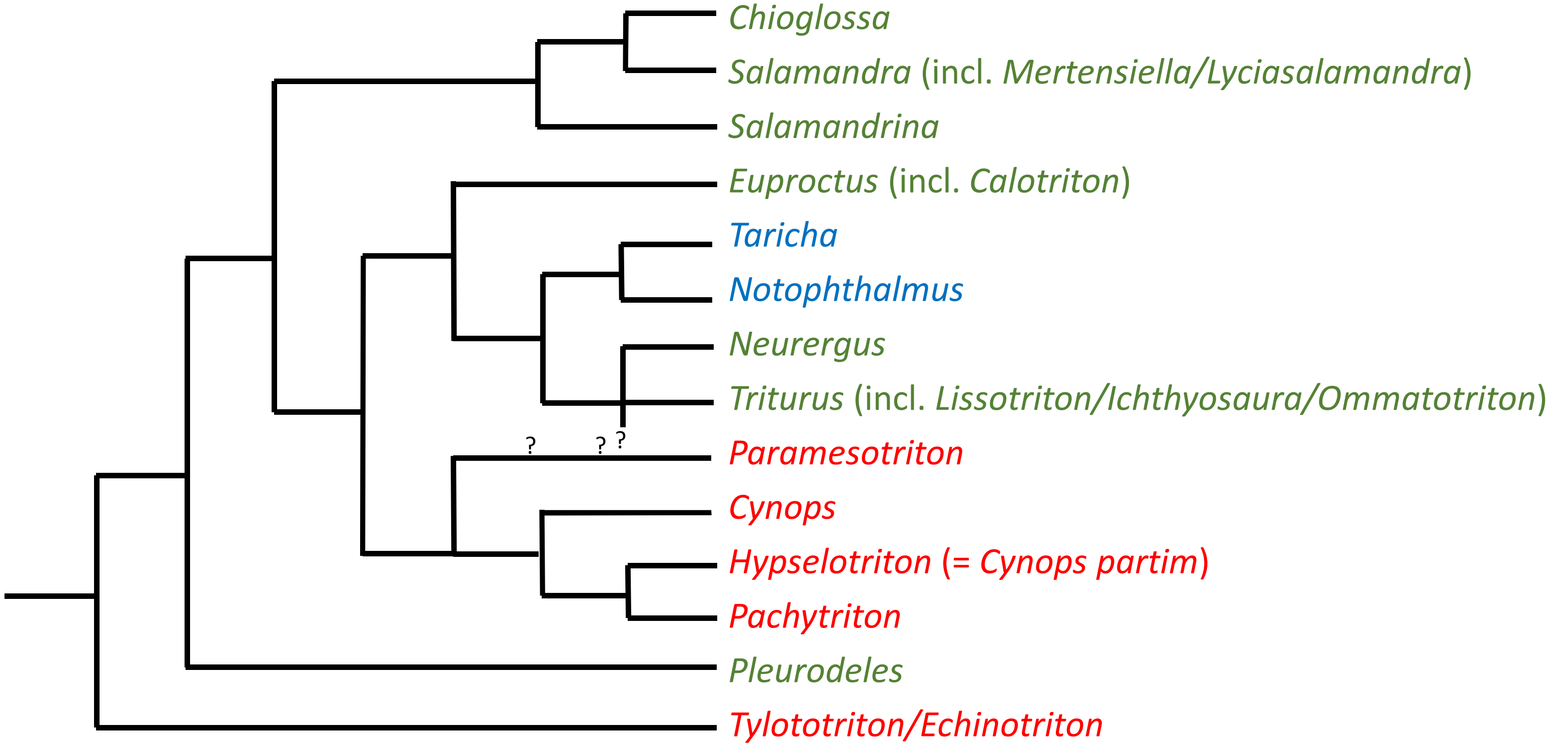

Wake and Özeti (1969):

- 27 characters;
  feeding mechanism
- Phenetic analysis; identification of plesiomorphic and apomorphic character states
- Final cladogram shown

Western Palearctic Salamandridae
Eastern Palearctic Salamandridae
Nearctic Salamandridae

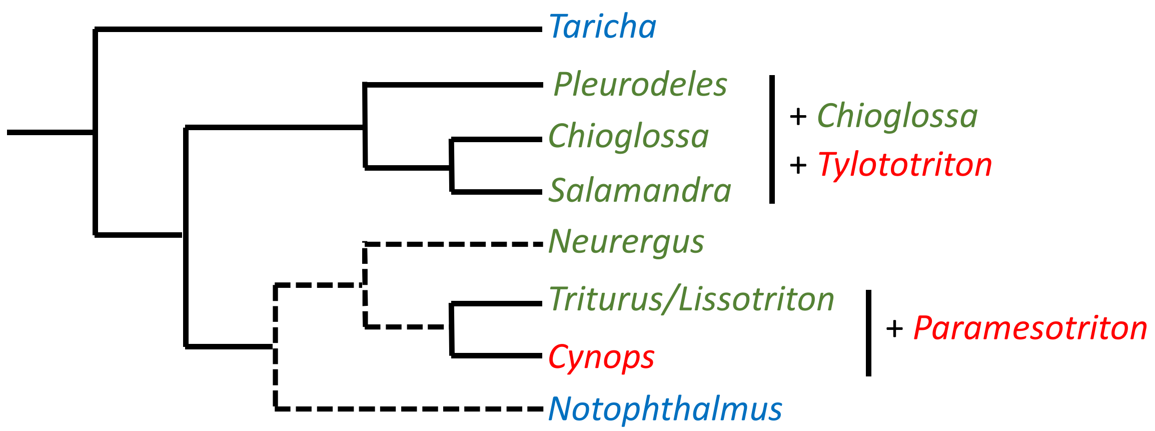

Arnold (1972):

- Behavioural characters
- Reconstruction based on his fig. 73 plus comments given in the text

Western Palearctic Salamandridae
Eastern Palearctic Salamandridae
Nearctic Salamandridae

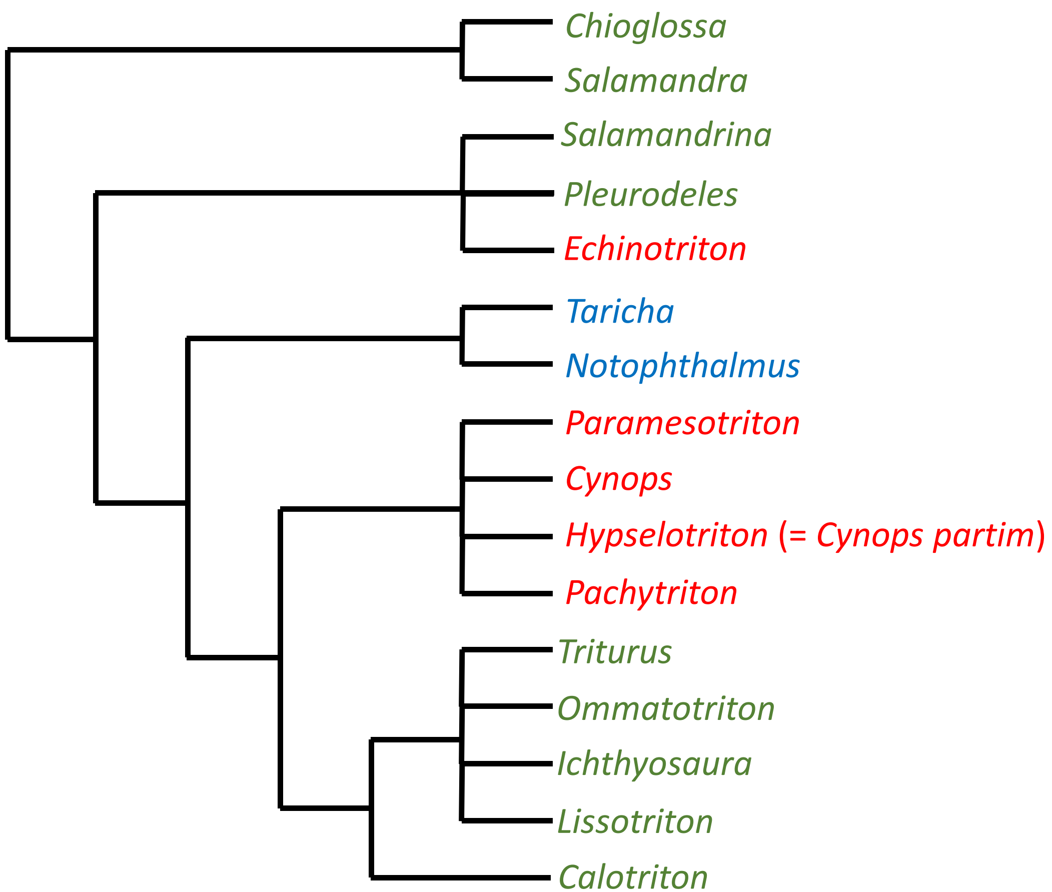

Naylor (1978):

- Vertebral column and trunk musculature
- Behavioural characters
- Further characters taken from literature
- Reconstruction based on his fig. 101 and comments given in the text

Western Palearctic Salamandridae
Eastern Palearctic Salamandridae
Nearctic Salamandridae

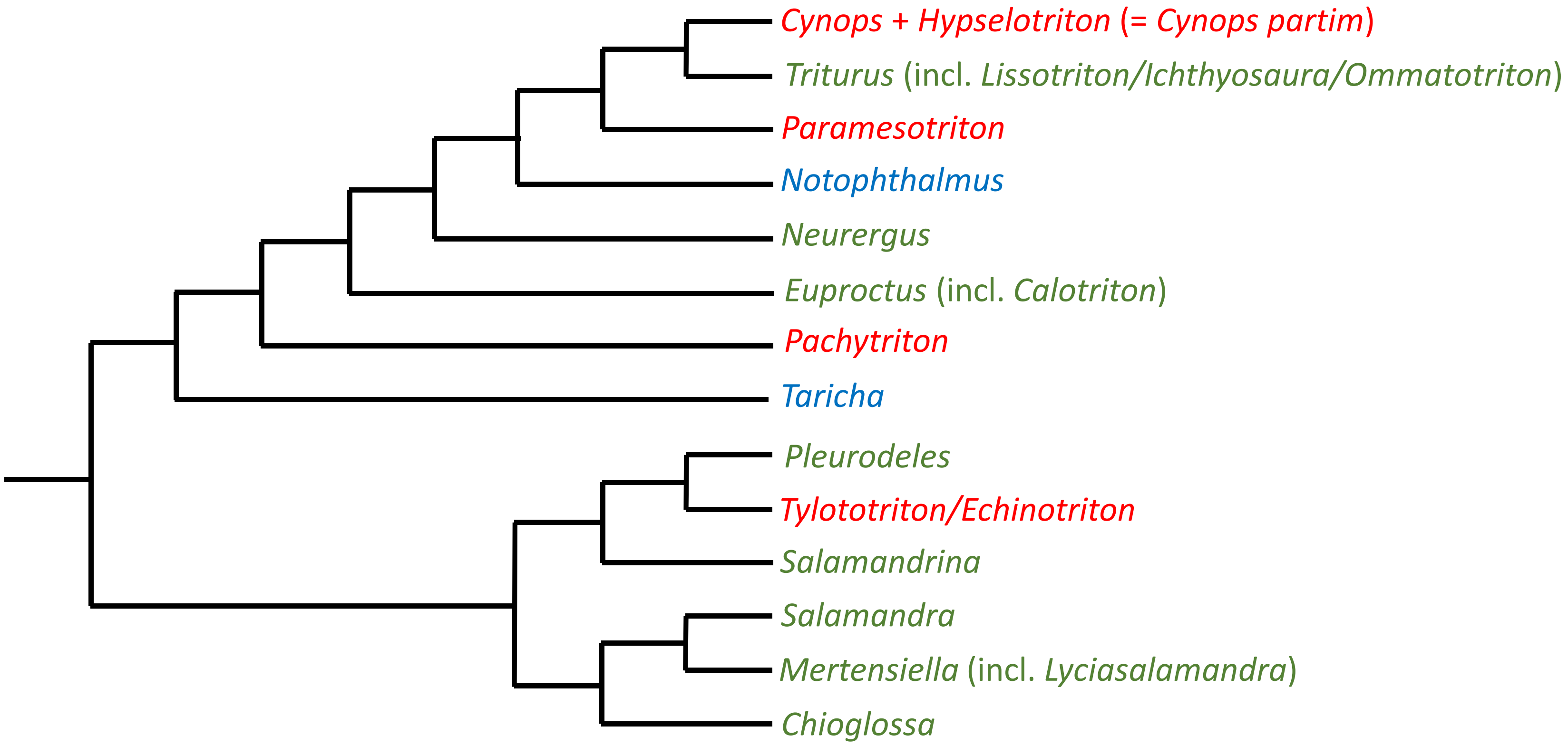

Freitag (1982):

- Morphology, life history and behaviour

Western Palearctic Salamandridae
Eastern Palearctic Salamandridae
Nearctic Salamandridae

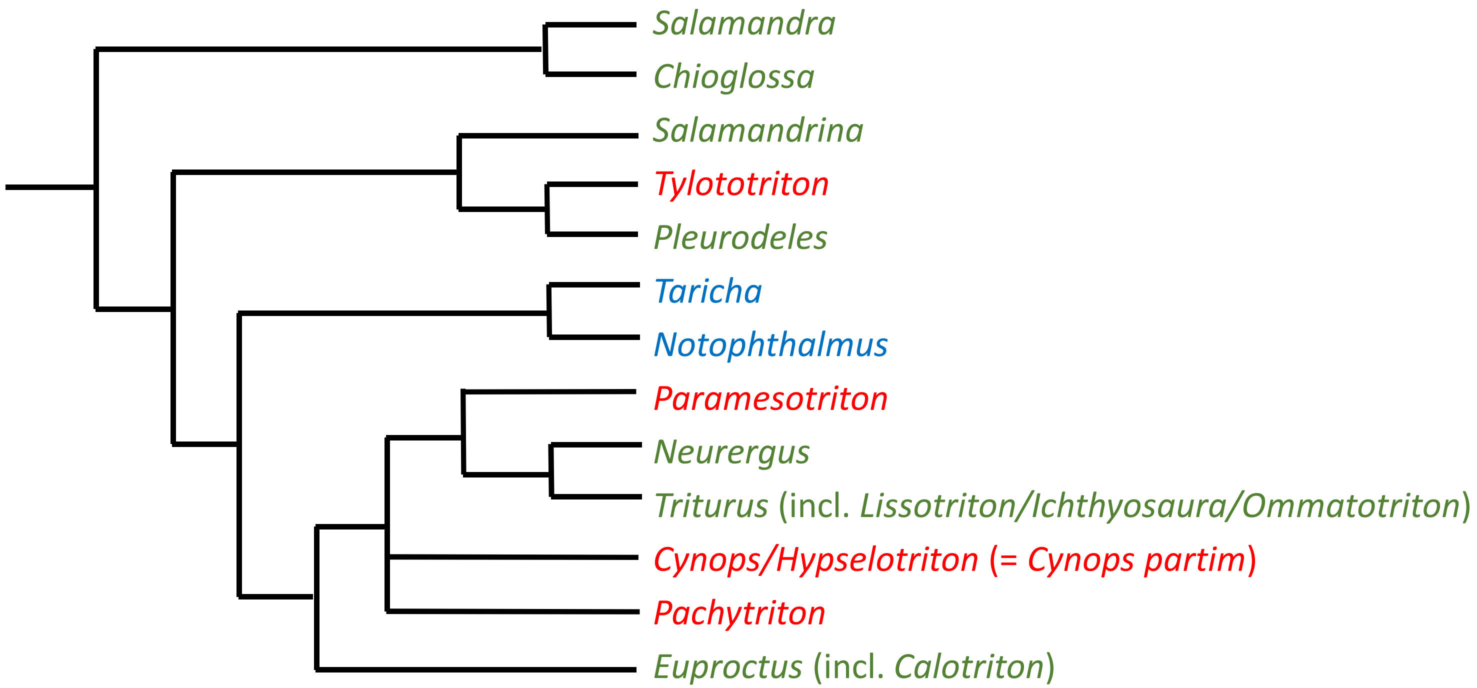

Laurent (1986):

- Osteology, dentition, outer morphology, mating behaviour, cytology
- Cladogram according to Scholz (1995)

Western Palearctic Salamandridae
Eastern Palearctic Salamandridae
Nearctic Salamandridae

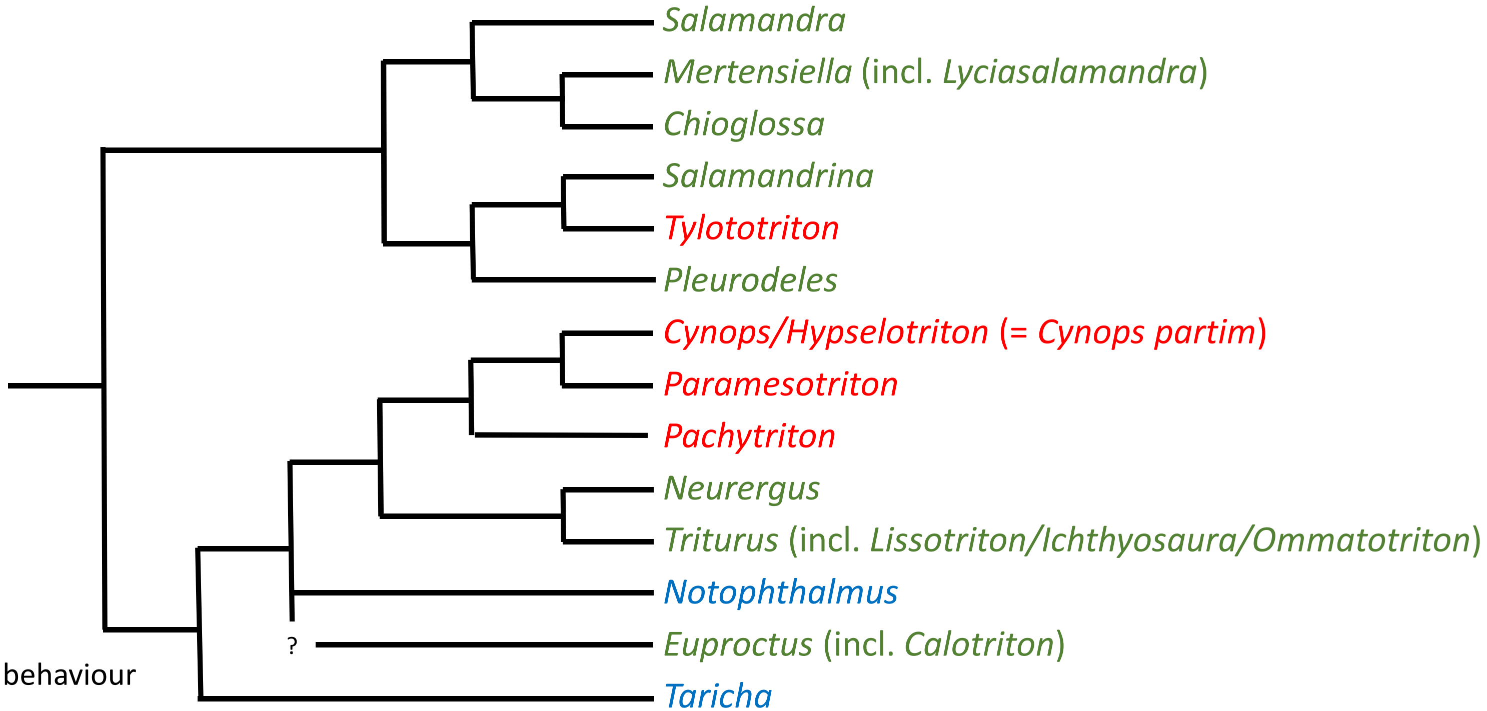

Scholz (1995): Cladogram I

- Osteology, external morphology and mating behaviour

Western Palearctic Salamandridae
Eastern Palearctic Salamandridae
Nearctic Salamandridae

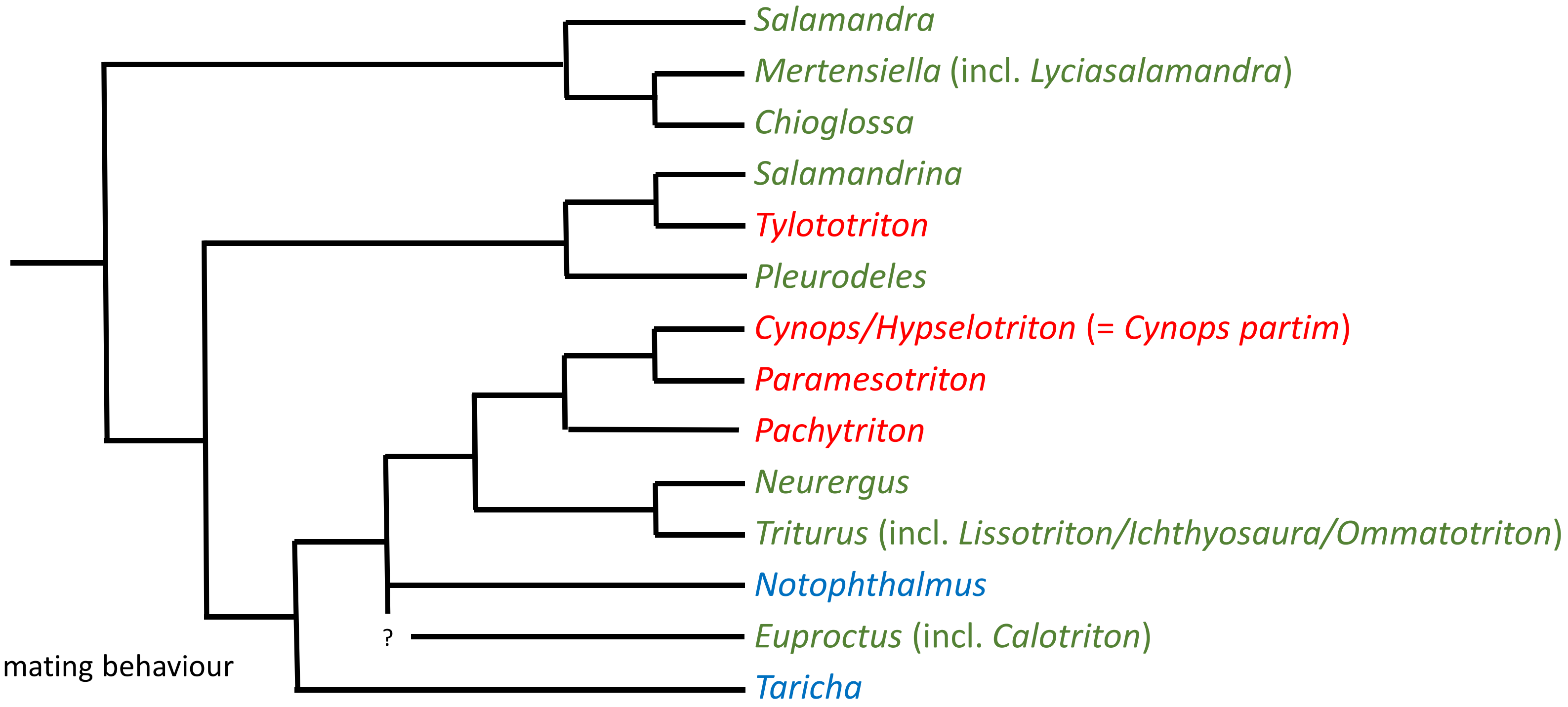

Scholz (1995): Cladogram II

- Osteology, external morphology and mating behaviour

Western Palearctic Salamandridae
Eastern Palearctic Salamandridae
Nearctic Salamandridae

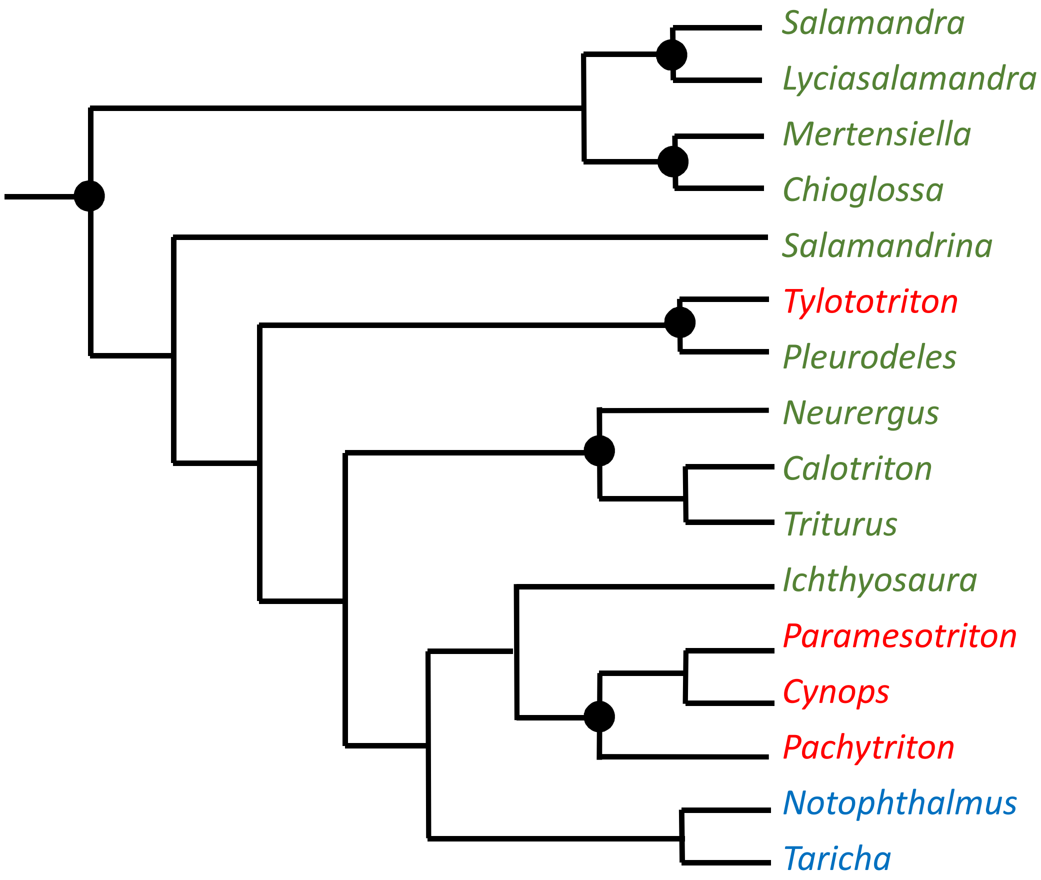

Titus and Larson (1995):

- Fragments of 12S and 16S, ca. 1,000 bp
- Maximum parsimony (their fig. 5)
- Black circles: nodes with bootstrap values ≥ 70%

Titus and Larson (1995):

- 1,000 bp mtDNA + 44 parsimony informative morphological characters
- Maximum parsimony
- No topology difference to the mtDNA tree

Western Palearctic Salamandridae
Eastern Palearctic Salamandridae
Nearctic Salamandridae
Other families

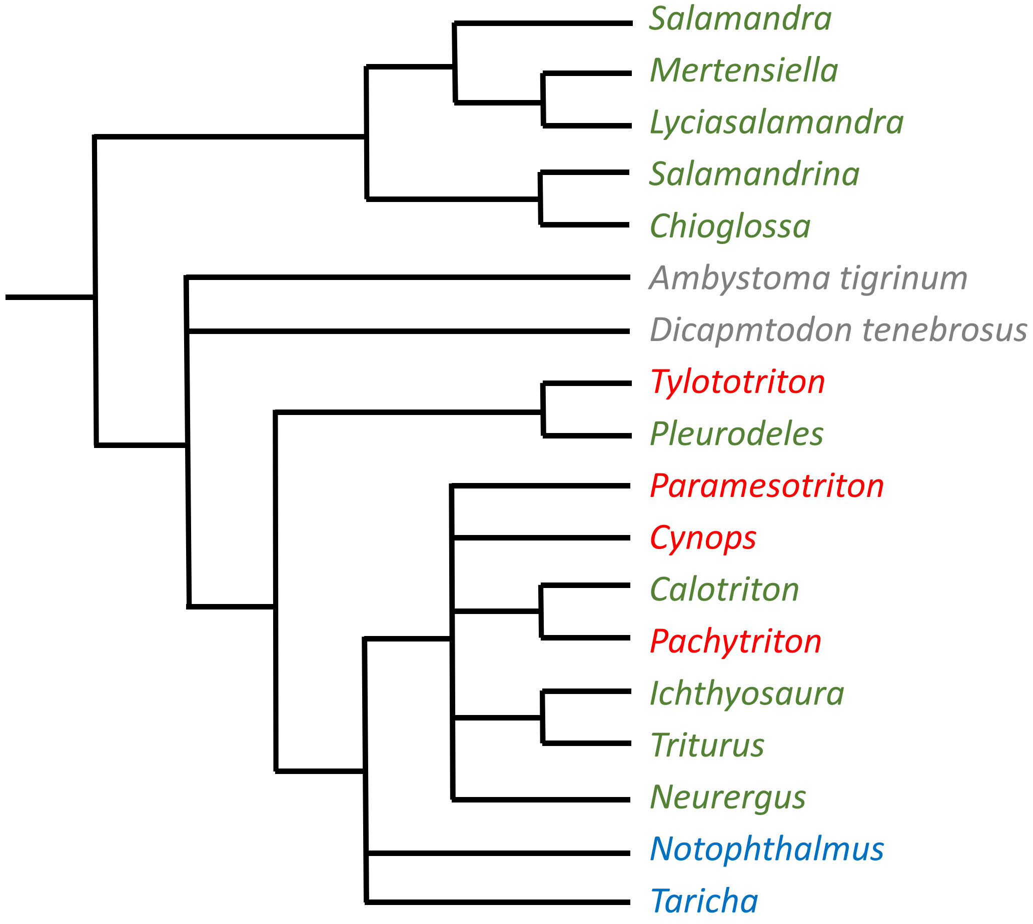

Titus and Larson (1995):

- 44 parsimony informative morphological characters

Western Palearctic Salamandridae
Eastern Palearctic Salamandridae
Nearctic Salamandridae

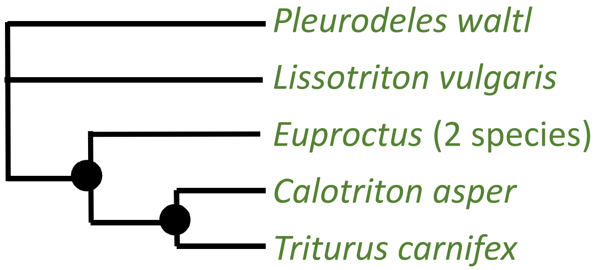

Caccone et al. (1997):

- MtDNA (fragments of 12S, 16S, cyt*b*); 1,582 bp
- Maximum parsimony
- Black circles: nodes with bootstrap values ≥ 70%

Western Palearctic Salamandridae
Eastern Palearctic Salamandridae
Nearctic Salamandridae

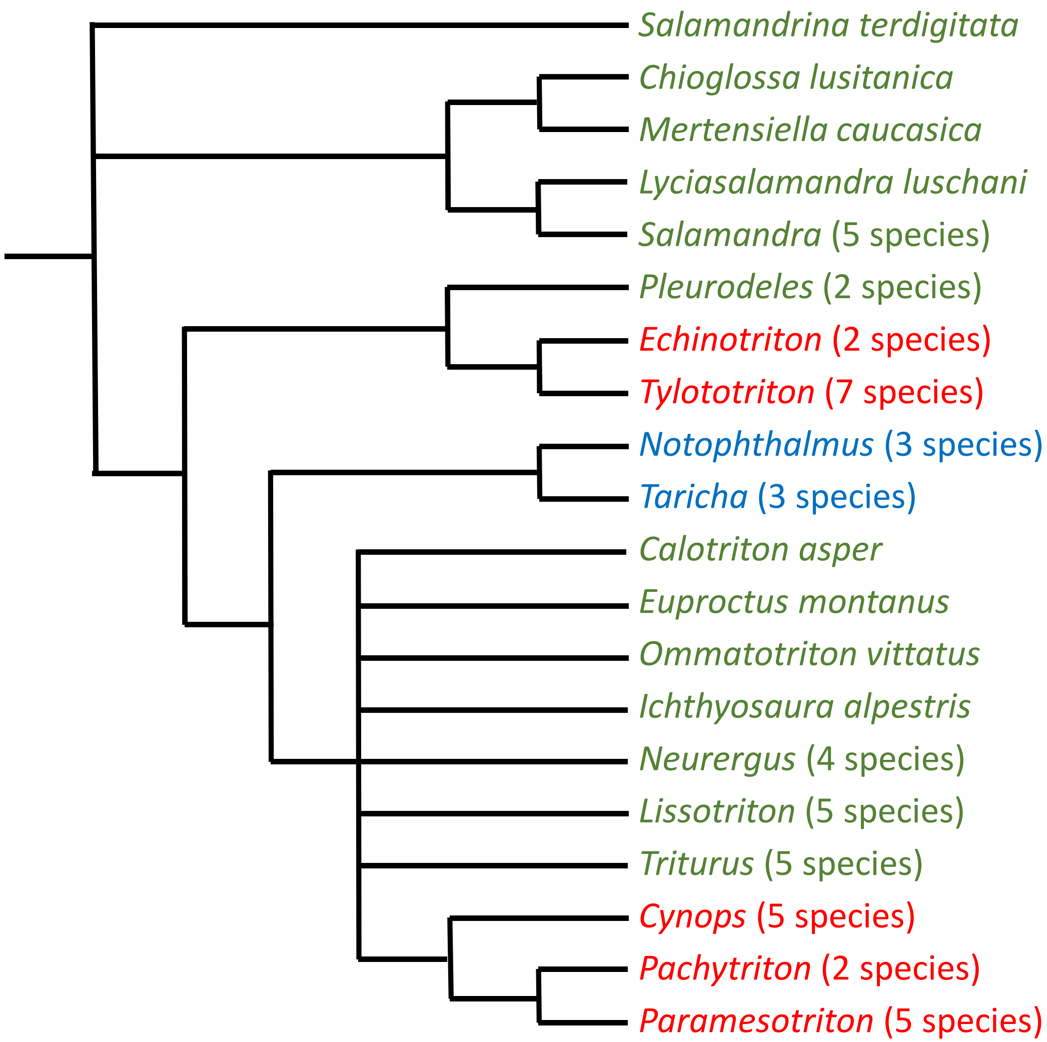

Sever et al. (2003):

- Consensus from existing evidence (allozymes, morphology, DNA data)

Western Palearctic Salamandridae
Eastern Palearctic Salamandridae
Nearctic Salamandridae

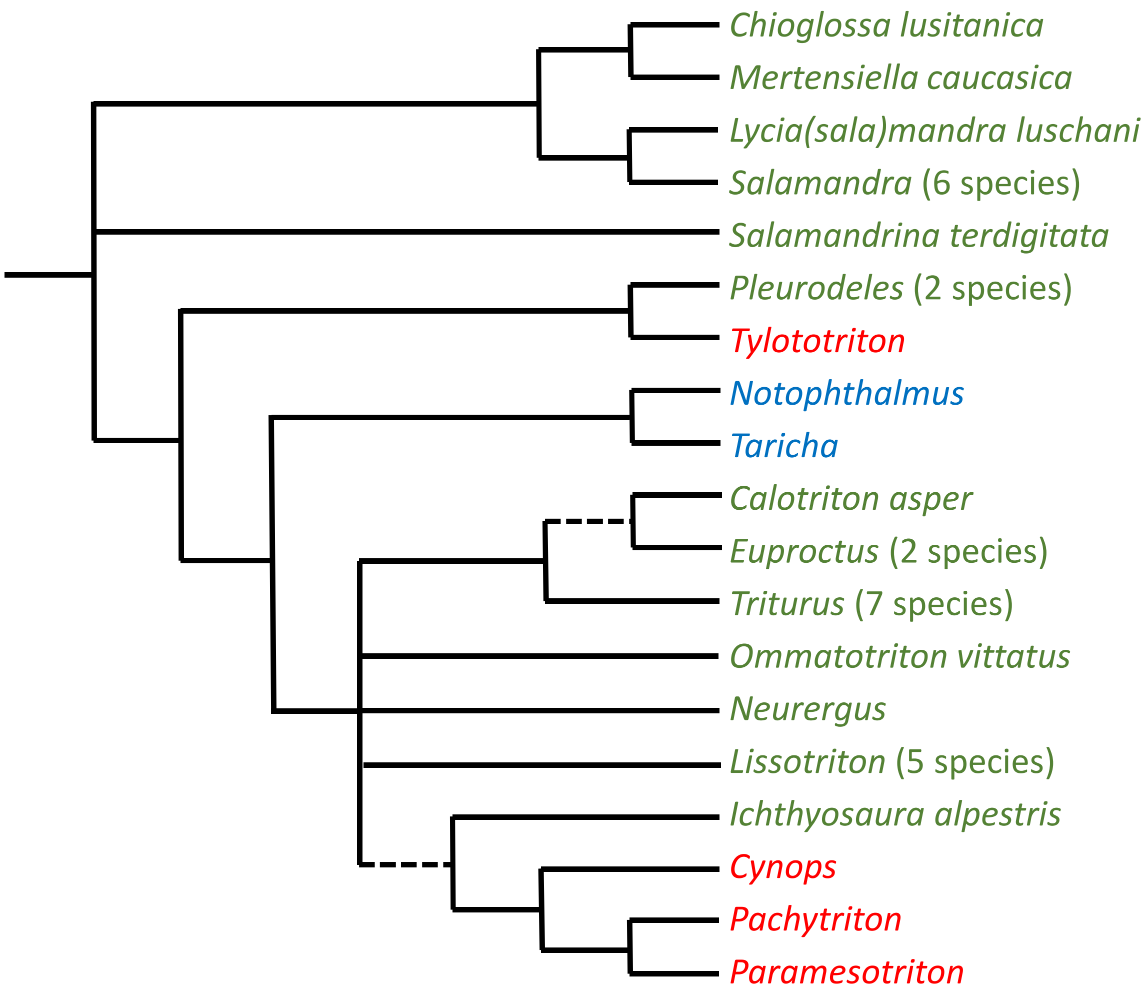

Garcia-Paris (2004):

- Consensus from existing evidence (molecular data)

Western Palearctic Salamandridae
Eastern Palearctic Salamandridae
Nearctic Salamandridae

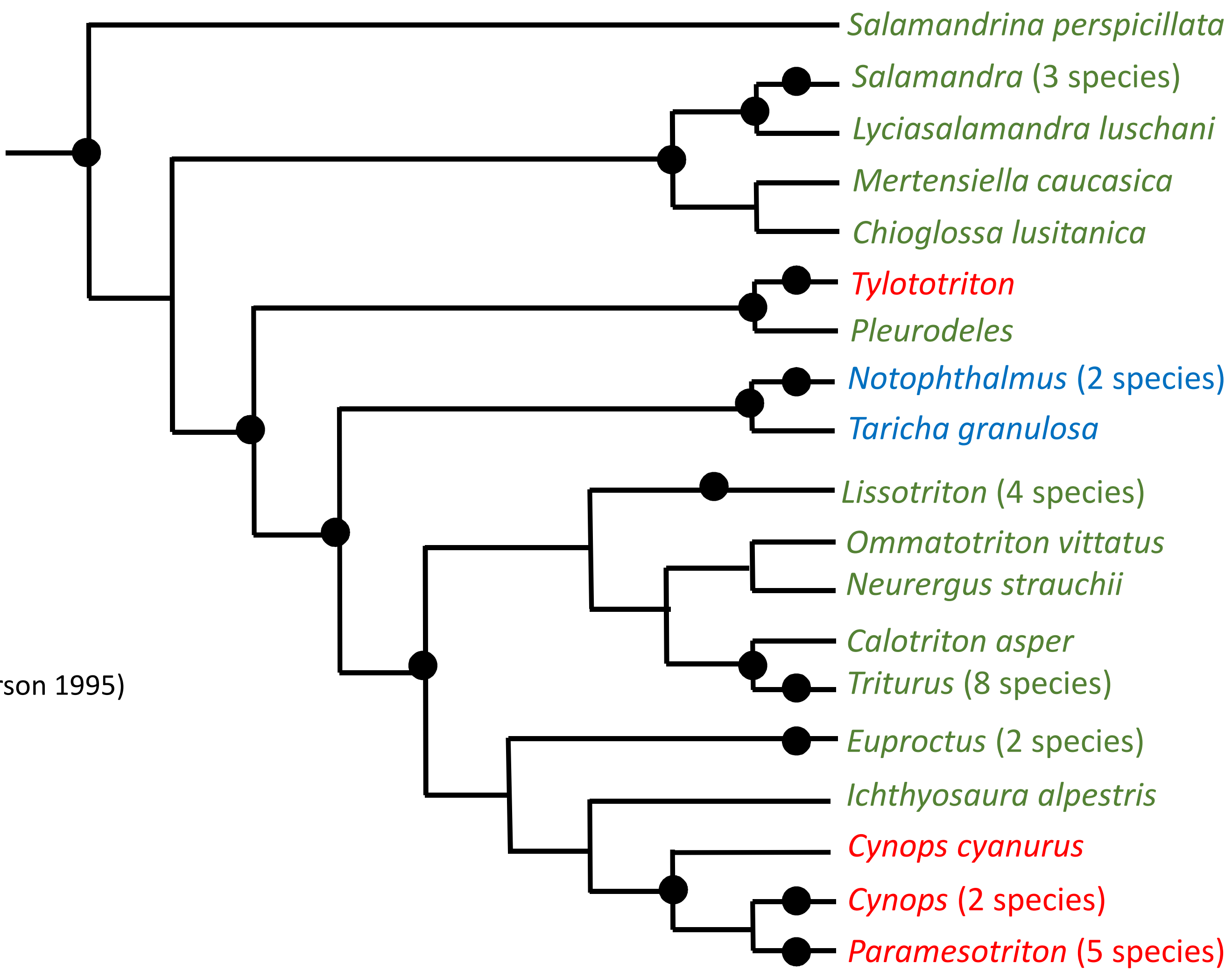

Steinfartz et al. (2006):

- 1,800 bp mtDNA (including the data of Titus and Larson 1995)
- Bayesian inference
- Black circles: nodes with BPP ≥ 95%

Western Palearctic Salamandridae
Eastern Palearctic Salamandridae
Nearctic Salamandridae

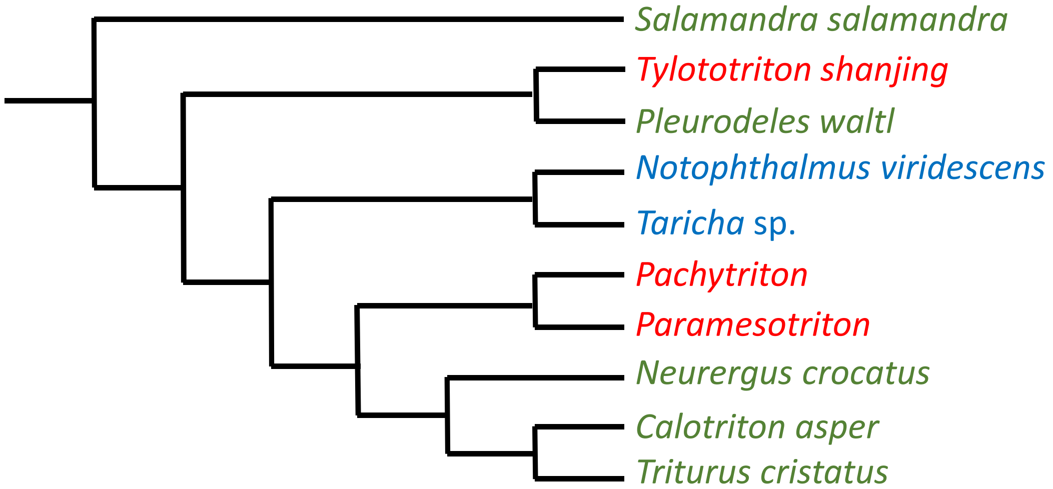

Frost et al. (2006):

- MtDNA (average: 2,400 bp) and ncDNA (average: 2,300 bp)
- POY analysis
- Bremer support and jackknife values are used,
  for which no reasonable thresholds can be defined.

Western Palearctic Salamandridae
Eastern Palearctic Salamandridae
Nearctic Salamandridae

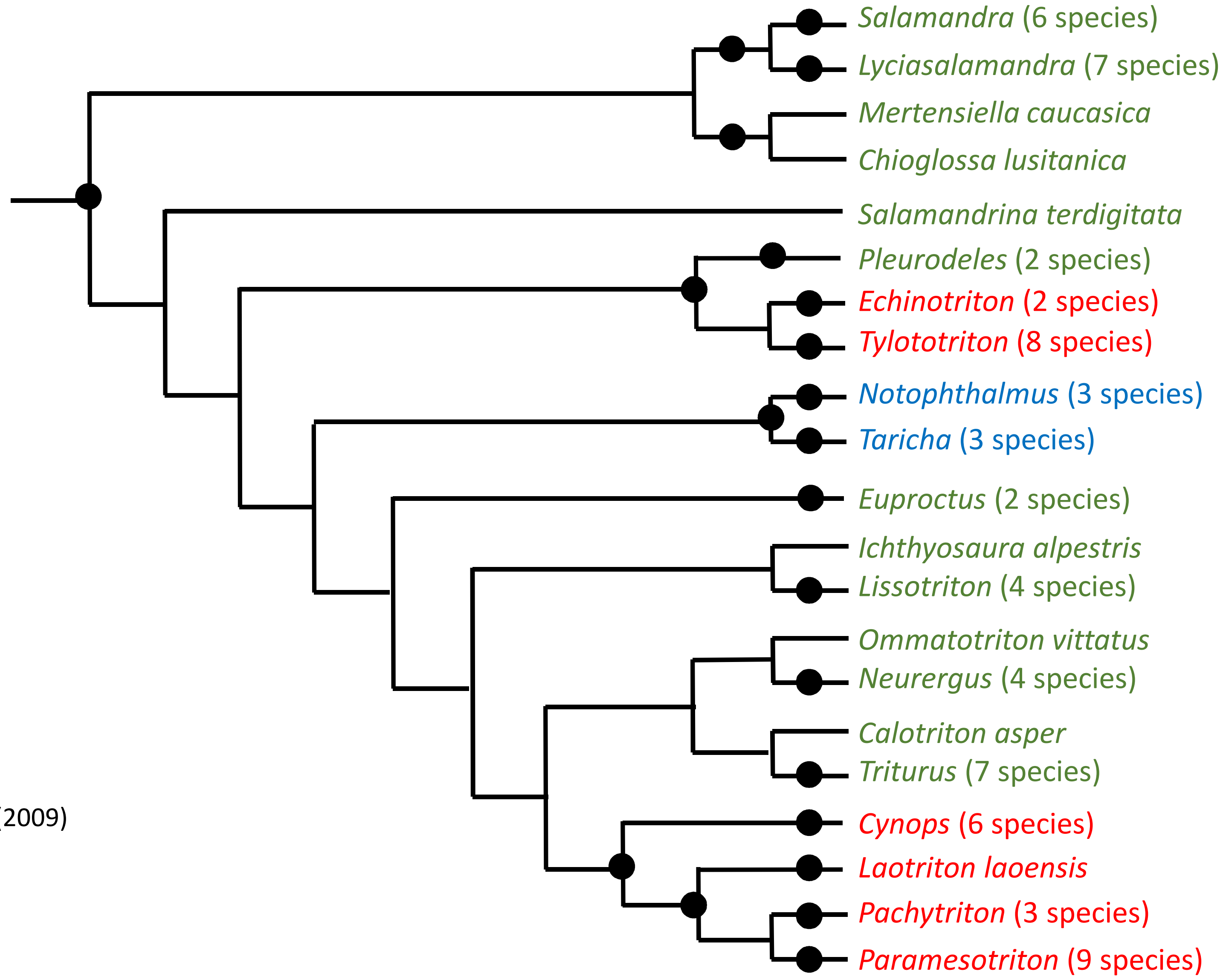

Weisrock et al. (2006):

- 2,700 bp mtDNA
- Bayesian inference
- Black circles: nodes with BPP ≥ 95%
- The same data set was used by Vieitis et al. (2009)

Western Palearctic Salamandridae
Eastern Palearctic Salamandridae
Nearctic Salamandridae

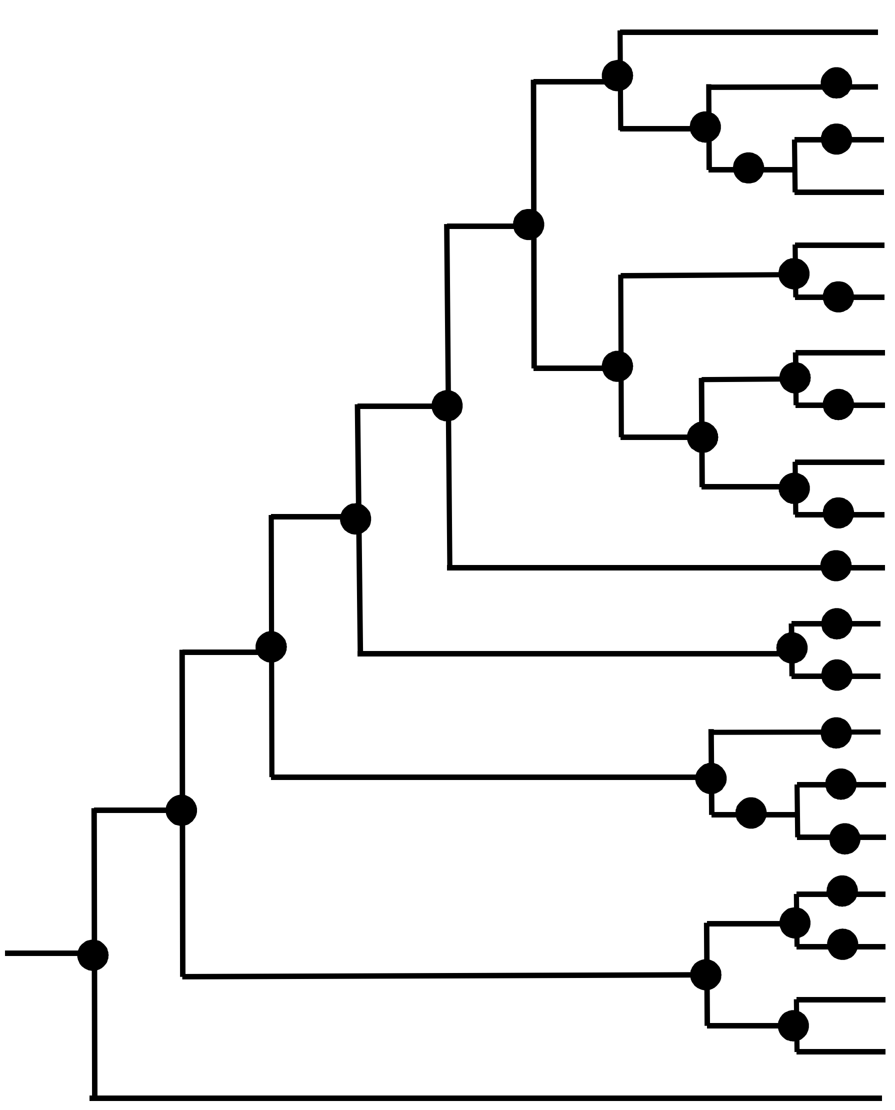

Zhang et al. (2008):

- Mitogenomes
- Bayesian inference
- Black circles: nodes with BPP ≥ 95%
- The tree infered by Kieren et al. (re-submitted) shows an identical topology since it used more or less the same data.

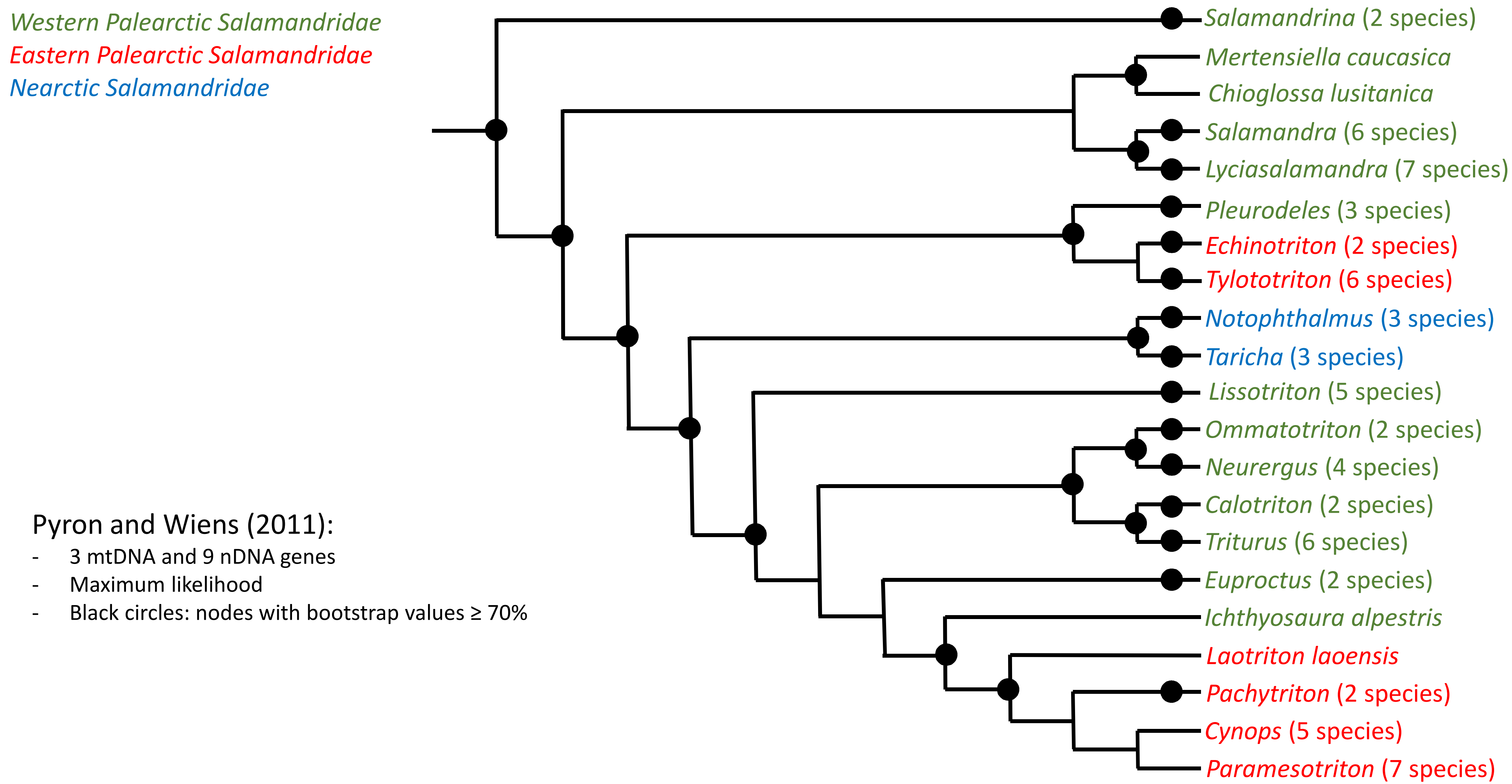

Pyron and Wiens (2011):

- 3 mtDNA and 9 nDNA genes
- Maximum likelihood
- Black circles: nodes with bootstrap values ≥ 70%

Western Palearctic Salamandridae
Eastern Palearctic Salamandridae
Nearctic Salamandridae

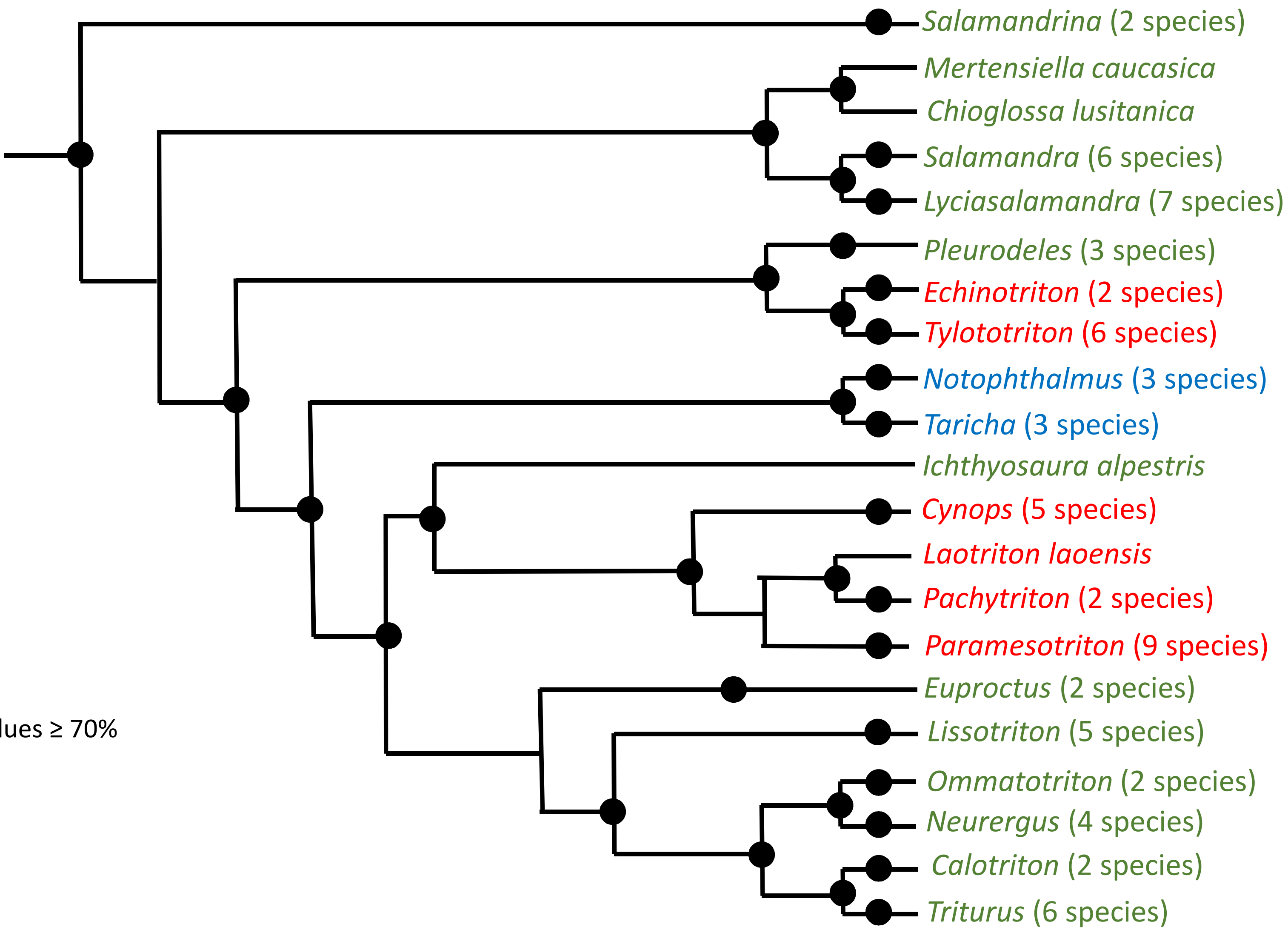

Pyron (2014):

- 3 mtDNA and 9 nDNA genes
- Maximum likelihood
- Black circles: nodes with bootstrap values ≥ 70%

Western Palearctic Salamandridae
Eastern Palearctic Salamandridae
Nearctic Salamandridae

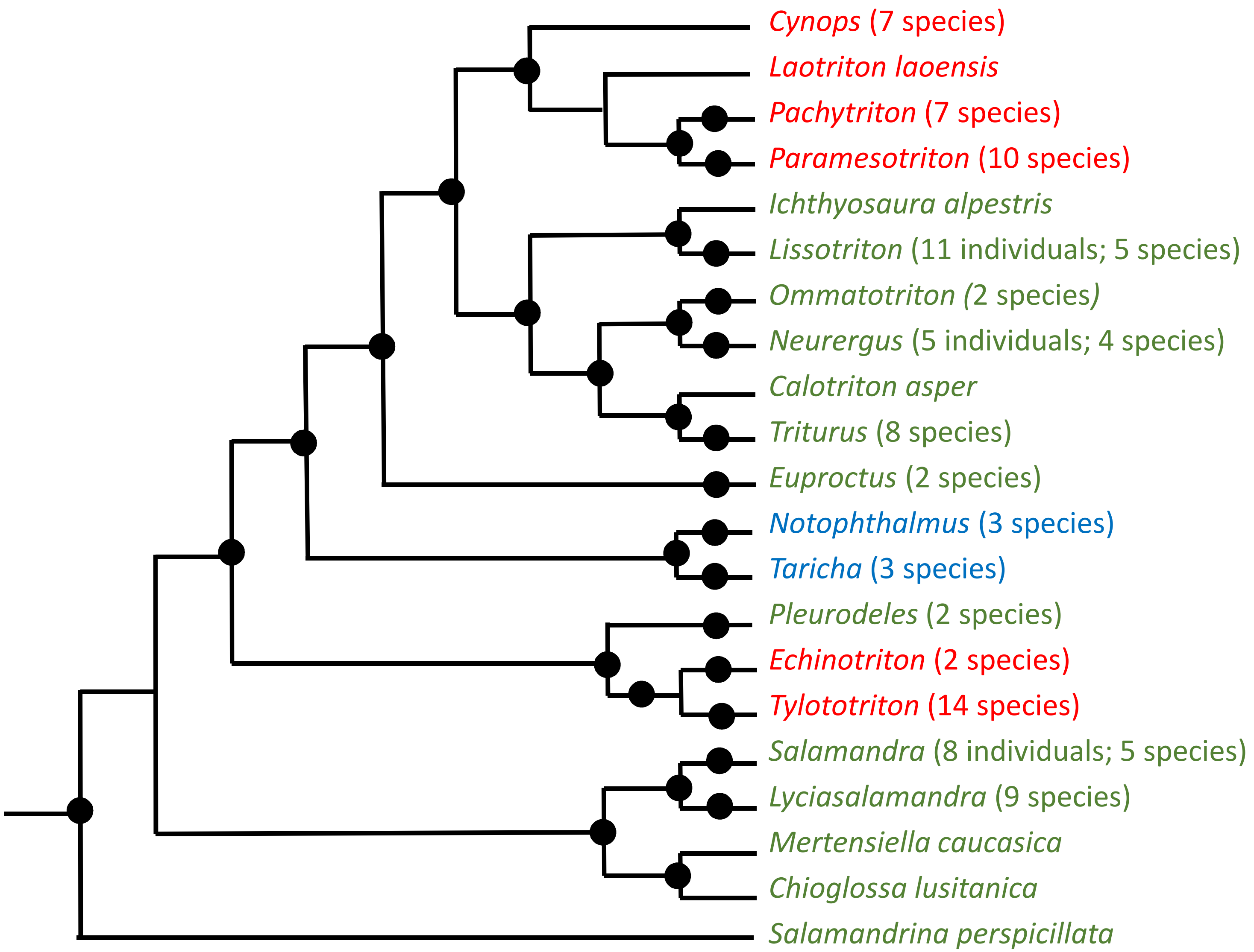

Arntzen et al. (2015):

- Mitogenomics (see Zhang et al. 2008) and sequences from ND1 to ND2 for additional taxa
- Bayesian inference
- Black circles: nodes with BPP ≥ 95%
- The genus *Cynops* does not appear as a monophylum

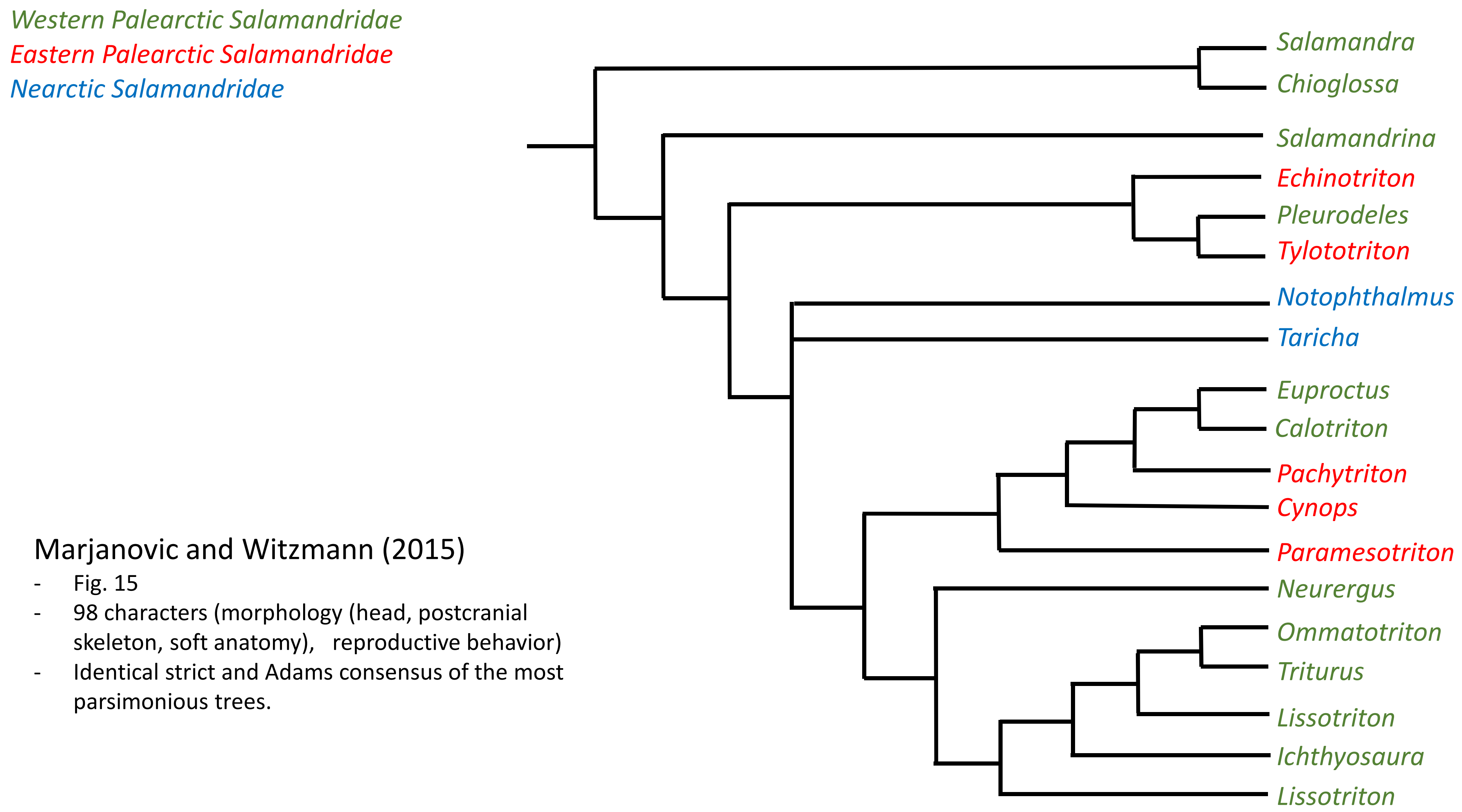

Marjanovic and Witzmann (2015)

- Fig. 15
- 98 characters (morphology (head, postcranial skeleton, soft anatomy), reproductive behavior)
- Identical strict and Adams consensus of the most parsimonious trees.

Western Palearctic Salamandridae
Eastern Palearctic Salamandridae
Nearctic Salamandridae

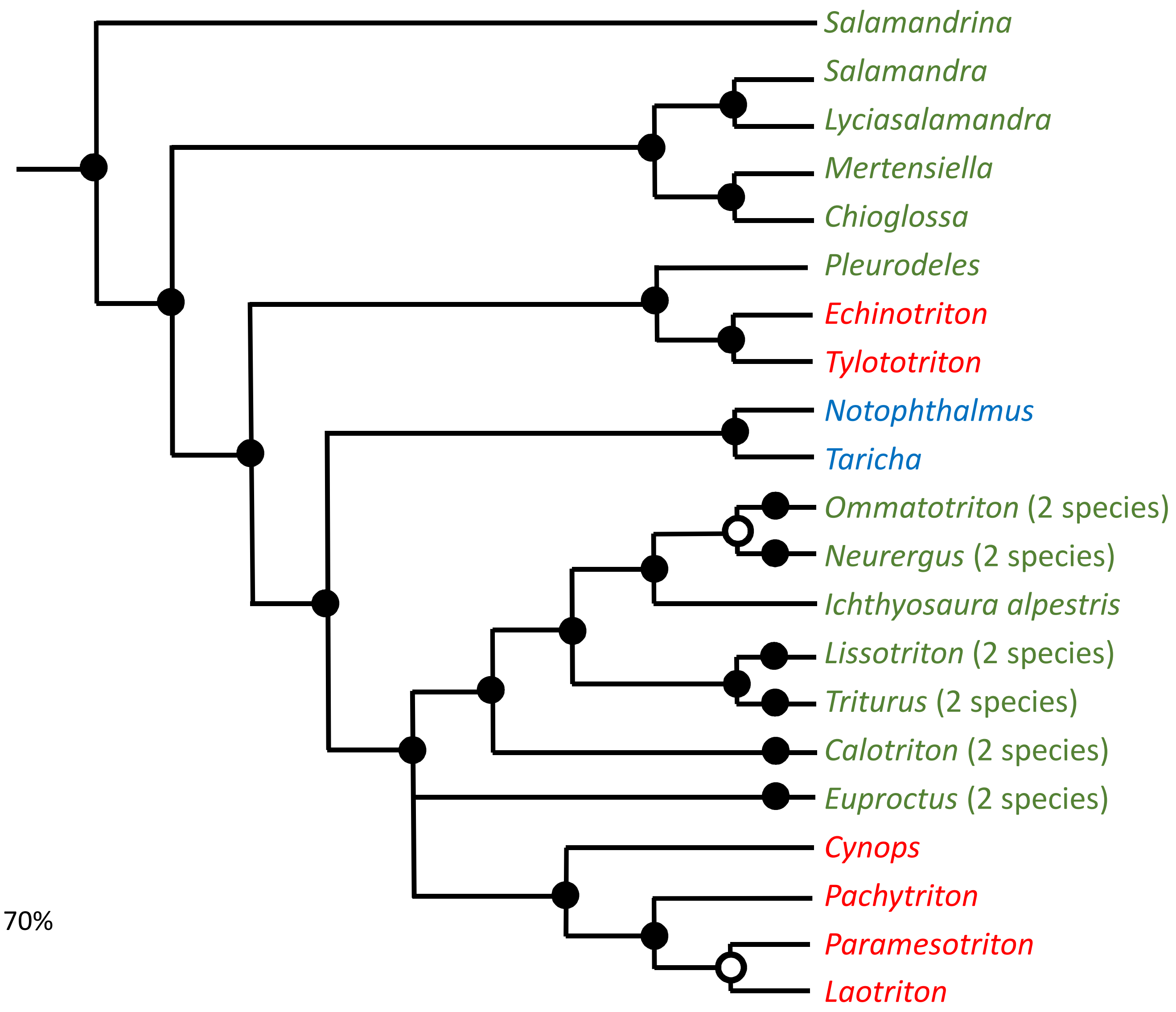

This paper:

- 4 nuclear genes
- Sometimes composite taxa
- Bayesian inference
- Black circles: nodes with BPP ≥ 95% and bootstrap support ≥ 70% in a maximum likelihood tree
- Open circles: nodes with only bootstrap support ≥ 70% in a maximum likelihood tree
