# Supplementary material for: The changing views on the evolutionary relationships of extant Salamandridae (Amphibia: Urodela)
Source: PLoS One. 2018 Aug 1;13(8):e0198237. doi: 10.1371/journal.pone.0198237 (PMC6070172; doi:10.1371/journal.pone.0198237)
Supplement: S1 File — (PDF) [file pone.0198237.s003.pdf]

S1 File. Phylogenetic inference of the four nuclear genes

To demonstrate the phylogenetic signal of each nuclear gene within the tree of the concatenated supermatrix, we reconstructed single gene trees: one tree for each nuclear gene. Analogous to the analysis in the main text, we used Partition Finder (version 2.1.1, Lanfear et al. 2016) to determine models of nucleotide evolution and the best-fitting partitioning scheme of subsets, implementing the greedy algorithm and the Akaike Information Criterion. We manually predefined three partitions for each gene, one partition for each single codon partition. We compared Partition Finder runs with unlinked and linked branch length and selected the best run according to the lowest AIC value; see results of Partition Finder in the table below. The results were used as a priori configurations in the phylogenetic tools.

Results of Partition Finder with predefined partitions and substitution models.

**Gene fragment**	**Predefined partitions**	**Substitution model**
Rag1	1st codon position	GTR+G
	2nd codon position	TVM+I+G
	3rd codon position	TVMef+G
	AIC value **linked 14076.72** *versus* unlinked 14171.61	
KIAA	1st codon position	TVMef+G
	2nd codon position	GTR+I+G
	3rd codon position	TVM+G
	AIC value **linked 8406.70** *versus* unlinked 8481.06	
SACS	1st codon position	K81uf+G
	2nd codon position	GTR+I
	3rd codon position	TrN+G
	AIC value **linked 8243.72** *versus* unlinked 8325.98	
TTN	1st codon position	TrN+I
	2nd codon position	TrNef+I+G
	3rd codon position	GTR+I
	AIC value **linked 6766.22** *versus* unlinked 6887.10	

Phylogenetic analyses were performed using Bayesian Inference (BI) (program MrBayes, version 3.2.6, Ronquist and Huelsenbeck 2003, Ronquist et al. 2012) and Maximum Likelihood (ML) (program RAxML, Stamatakis 2014). For the BI, we started two independent runs to avoid local maxima; each analysis runs four independent MCMC analyses with one cold and three heated chains for 10 million generations and was sampled every $1,000^{th}$ generations with a burn in of 20%. The ML tree was calculated with 2,000 bootstrap replicates using rapid bootstrapping and the greedy algorithm. The phylogenetic trees of the four gene fragments are shown below.

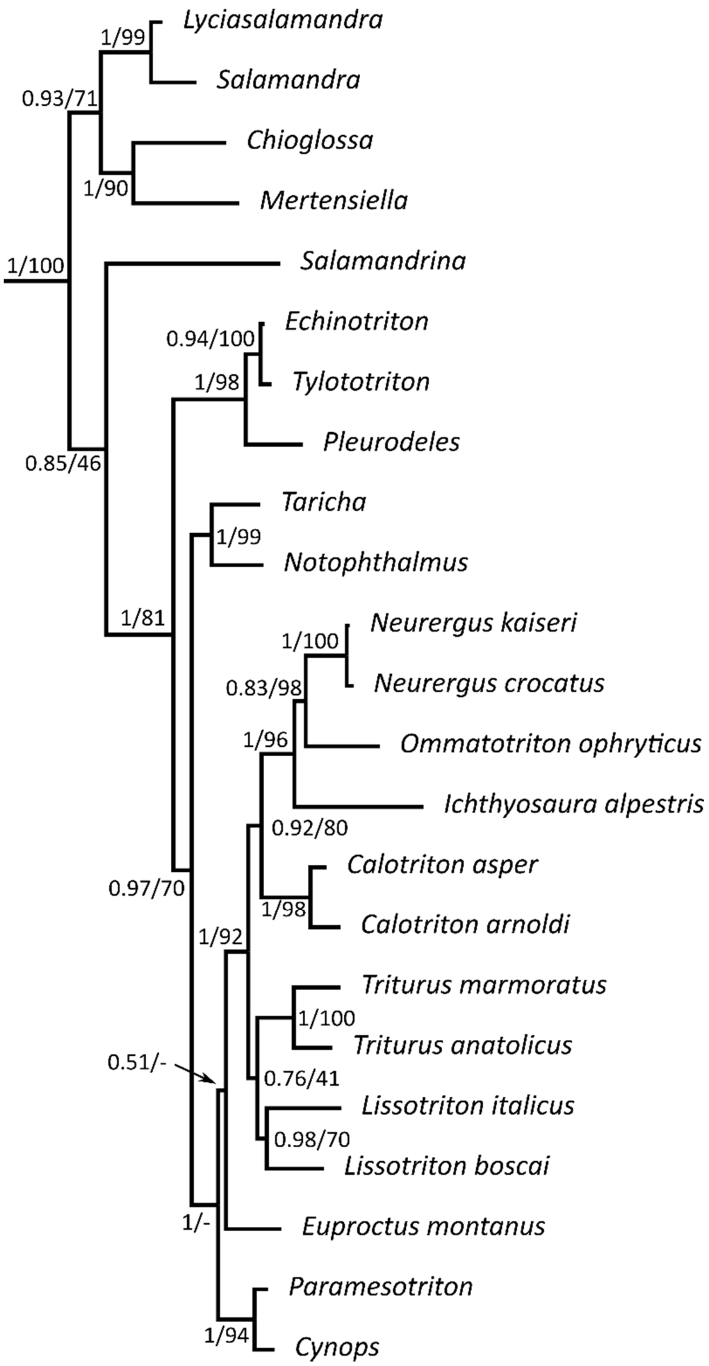

Bayesian Inference tree of Salamandridae based on the nuclear gene fragment Rag1.

Values on nodes represent Bayesian posterior probabilities/bootstrap values. Missing sequence information: *Pachytriton*, *Laotriton*, *Euproctus platycephalus* and *Ommatotriton vittatus*.

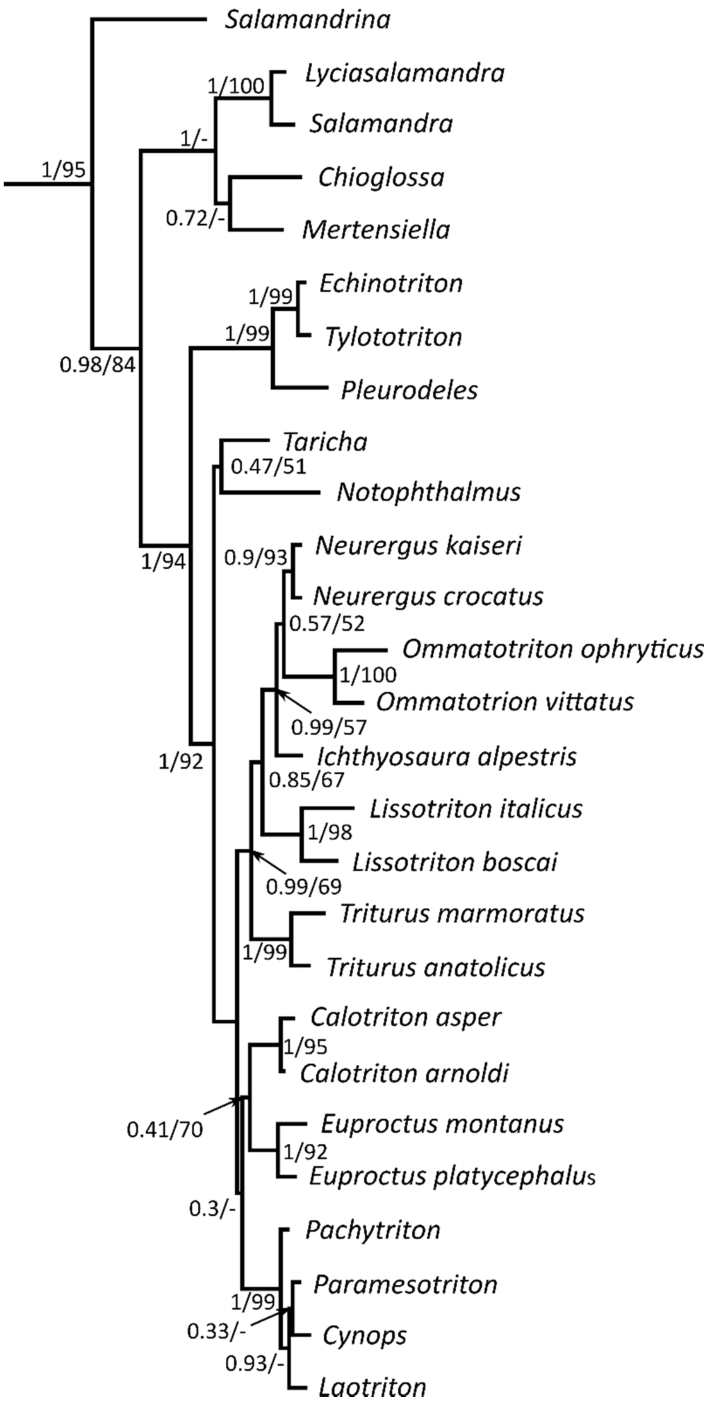

Bayesian Inference tree of Salamandridae based on the nuclear gene fragment KIAA.

Values on nodes represent Bayesian posterior probabilities/bootstrap values.

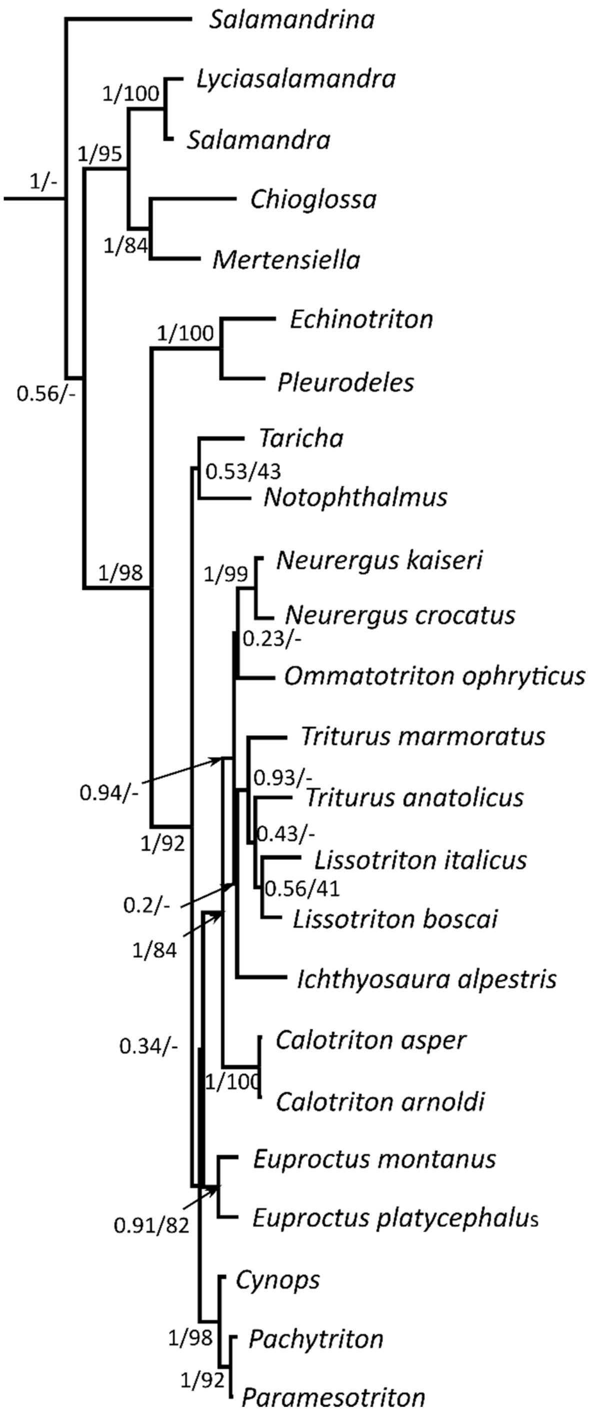

Bayesian Inference tree of Salamandridae based on the nuclear gene fragment SACS.

Values on nodes represent Bayesian posterior probabilities/bootstrap values. Missing sequence information: *Ommatotriton vittatus*, *Tylototriton* and *Laotriton*.

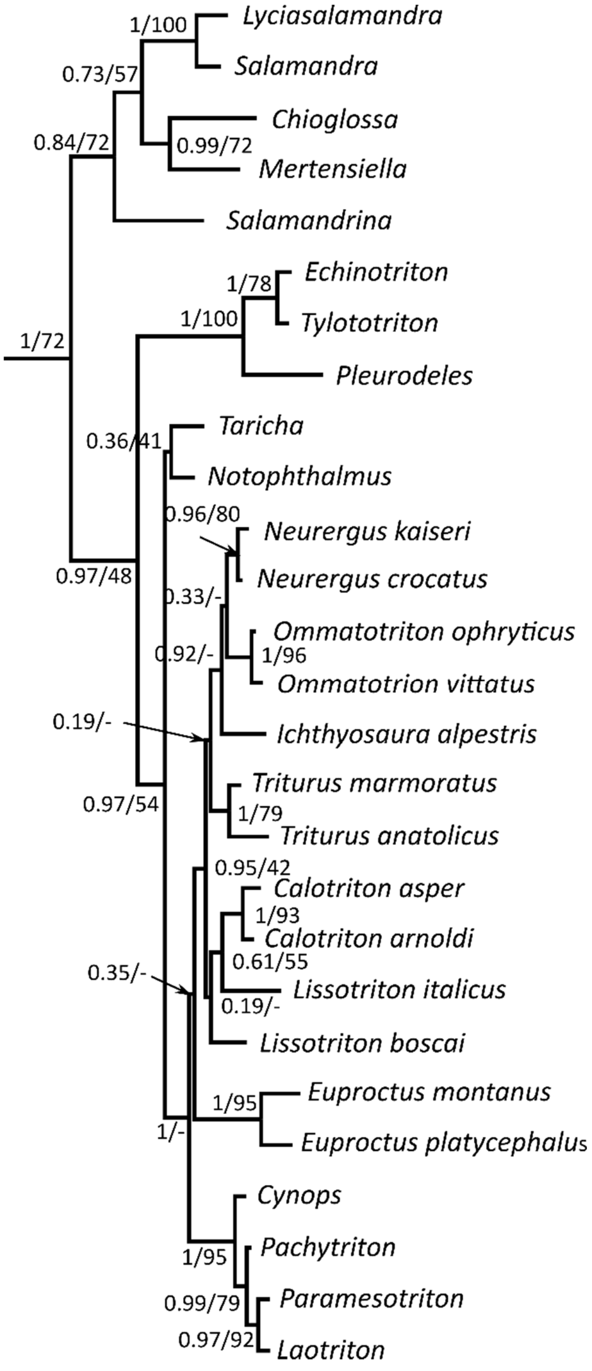

Bayesian Inference tree of Salamandridae based on the nuclear gene fragment TTN.

Values on nodes represent Bayesian posterior probabilities/bootstrap values.

References

Lanfear, R., Frandsen, P. B., Wrigt, A. M., Senfeld, T., Calcott, B. (2016): PartitionFinder2: new methods for selecting partitioned models of evolution for molecular and morphological phylogenetic analyses. Mol. Biol. Evol., 34: 772-773.

Ronquist, F., Huelsenbeck, J.P (2003): MrBayes 3: Bayesian phylogenetic inference under mixed models. Bioinformatics, 19: 1572-1574.

Ronquist, F., Teslenko, M., van der Mark, P., Ayres, D.L., Darling, A., Höhna, S., Larget, B., Liu, L., Suchard, M.A., Huelsenbeck, J.P. (2012): MrBayes 3.2: Efficient Bayesian phylogenetic inference and model choice across a large model space. Syst. Biol., 61: 539-542.

Stamatakis, A. (2014): RAxML version 8: A tool for phylogenetic analysis and post-analysis of large phylogenies. Bioinformatics, 30: 1312-1313.
